# Piezo1 ion channel pore properties are dictated by C-terminal region

**DOI:** 10.1038/ncomms8223

**Published:** 2015-05-26

**Authors:** Bertrand Coste, Swetha E. Murthy, Jayanti Mathur, Manuela Schmidt, Yasmine Mechioukhi, Patrick Delmas, Ardem Patapoutian

**Affiliations:** 1 Aix Marseille Université, CNRS, CRN2M-UMR7286, 13344 Marseille, France; 2 Howard Hughes Medical Institute, Molecular and Cellular Neuroscience, The Scripps Research Institute, La Jolla, California 92037, USA; 3 Genomics Institute of the Novartis Research Foundation, San Diego, California 92121, USA

## Abstract

Piezo1 and Piezo2 encode mechanically activated cation channels that function as mechanotransducers involved in vascular system development and touch sensing, respectively. Structural features of Piezos remain unknown. Mouse Piezo1 is bioinformatically predicted to have 30–40 transmembrane (TM) domains. Here, we find that nine of the putative inter-transmembrane regions are accessible from the extracellular side. We use chimeras between mPiezo1 and dPiezo to show that ion-permeation properties are conferred by C-terminal region. We further identify a glutamate residue within a conserved region adjacent to the last two putative TM domains of the protein, that when mutated, affects unitary conductance and ion selectivity, and modulates pore block. We propose that this amino acid is either in the pore or closely associates with the pore. Our results describe important structural motifs of this channel family and lay the groundwork for a mechanistic understanding of how Piezos are mechanically gated and conduct ions.

## Abstract (web summary)

Piezo ion channels function as mechanotransducers involved in vascular development and touch sensing, but their structural features remain unknown. Here the authors find that the C-terminal region of Piezo protein encompasses the pore and identify a glutamate residue within this region involved in ion conduction properties.

## Graphical abstract

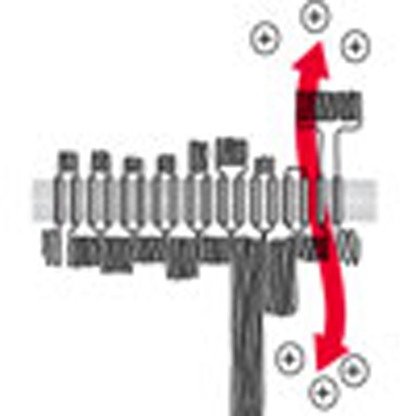

Mechanotransduction is the process by which mechanical stimuli are converted into biological activity. Piezos are mechanically activated (MA) cation channels conserved through evolution and act as mechanotransducers in various biological processes. The single Piezo gene in flies is involved in nociception^1^; zebrafish and mouse Piezo2 in touch sensation^2^,^3^,^4^,^5^,^6^; zebrafish Piezo1 in red blood cell volume regulation^7^; and mouse Piezo1 in vascular development^8^,^9^. In humans, mutations that alter channel gating of Piezo1 and 2 are linked to various disorders with dominant inheritance^10^,^11^,^12^. Piezo proteins contain over 2,000 amino acid residues with an estimated 30–40 transmembrane (TM) segments and are likely to form homo-tetramers in a complex weighing over 1.2 million daltons^13^,^14^. Piezos lack homology with other proteins, and their structural features remain unknown. The large size and numerous hydrophobic domains of Piezos constitute technical challenges for structural analysis of the intact channel^15^. Basic questions regarding Piezo topology and the location of the ion-permeation pathway remain unanswered, and yet these questions are crucial for a mechanistic understanding of how the channel is gated by mechanical forces, and how human disease-related point mutations affect channel function^16^.

We aimed to determine the topology of these large proteins and delineate the amino-acid residues involved in the ion-permeation pathway. Here, we provide experimental evidence to confirm the position of the amino (N)- and carboxy (C)-terminals and 13 of the putative inter-hydrophobic loop regions of the protein. We show that the C-terminal region of the Piezo protein encompasses the pore. Within this region, we identify a glutamate residue involved in ion conduction properties. Our results lay the groundwork for understanding how Piezos are mechanically gated and conduct ions, and how Piezo mutations affect human biology.

## Results

### Transmembrane topology of Piezo channels

**Figure 1:**
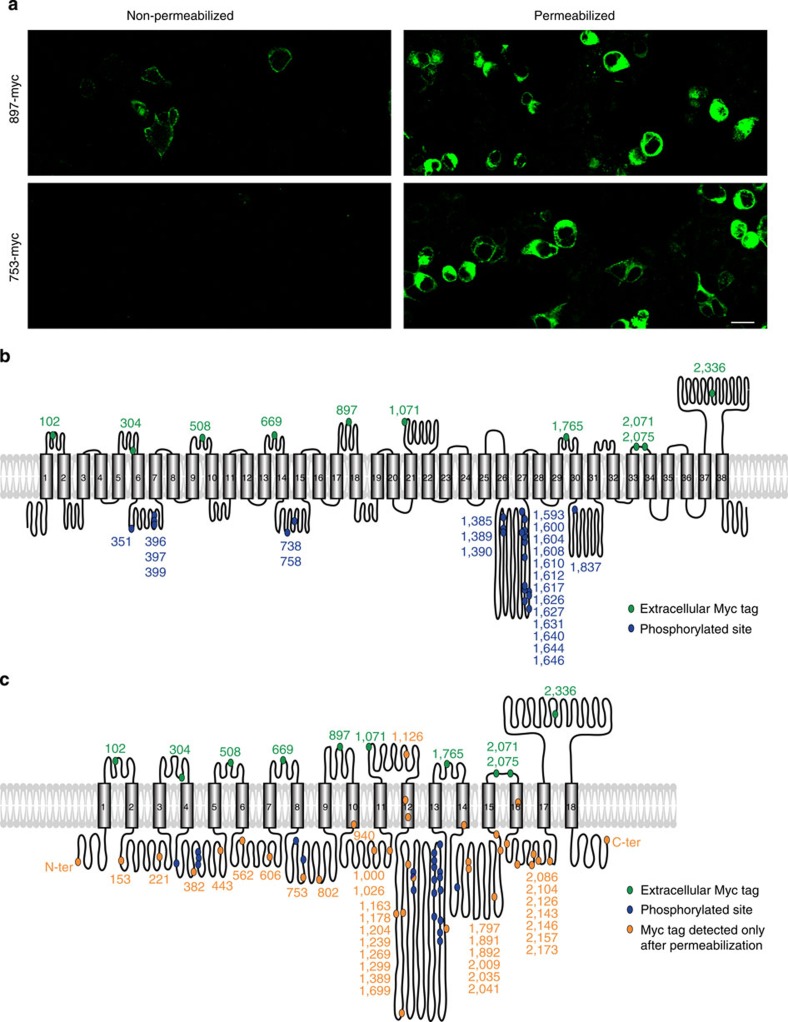
Transmembrane topology of mPiezo1. (**a**) Representative images (three experimental replicates) of Myc labelling in mPiezo1-Myc transfected HEK293T cells. Myc tags were inserted at position 897 (upper panels) or 753 (lower panels). Immunostaining was performed prior (left panels) or after (right panels) cell permeabilization. Scale bar, 20 μm. (**b**,**c**) Schematic of transmembrane topology of mPiezo1 protein showing positions of Myc tags detected extracellularly (green) and phosphorylated sites detected by mass spectrometry (blue). (**c**) Positions of Myc tags detected only after permeabilization (orange) are shown.

To characterize the transmembrane topology of mPiezo1 we combined bioinformatics analysis, immunostaining to detect extracellular tags inserted in predicted loop regions, and detection of intracellular phosphorylation sites by mass spectrometry (Fig. 1). First, we used hydrophobicity plots, transmembrane segment predictions and sequence alignment of functionally tested Piezo proteins (human, mouse and fly Piezos) to generate a predicted transmembrane topology of mPiezo1. This virtual topology was then tested experimentally by inserting Myc tags at mPiezo1 termini and in each predicted loop. In some cases, more than one tag was tested per predicted loop. Each of these constructs was subjected to an immunostaining protocol to test whether an anti-Myc antibody could recognize the Myc epitope in mPiezo1 in non-permeabilized cells, suggesting an extracellular topology (Fig. 1a and Supplementary Fig. 1). A negative signal would suggest an intracellular epitope or one that is masked by the cell membrane or another such mechanism^17^. Therefore, only tags that gave a positive signal were used to predict the topology. A total of 48 Myc constructs were designed. Forty-five of these constructs showed staining with the Myc antibody after permeabilization, among which only 10 constructs were positive for staining without permeabilization, and therefore predicted to be present extracellularly (Supplementary Fig. 2 and Supplementary Table 1). Two among the 10 Myc constructs were four amino acids apart and can account only for a single extracellular loop, therefore nine extracellular loops are labelled. The orientation of these extracellular loops agreed with the hydropathy/hydrophilicity plot predictions. Insertion of Myc tags at the N- or C-terminal, and at seven out of the nine extracellular loop positions did not affect channel function, and resulted in MA currents of amplitude comparable to WT currents. Whole-cell currents could not be detected from constructs with Myc tags at amino acid position 2,071, 2,075 or 2,336, suggesting that tag insertions there compromised channel function. Next, using mass spectrometry, we analysed phosphorylated sites within mPiezo1 purified protein to identify intracellular loops in the protein. A total of 24 phosphorylated peptides were detected corresponding to phosphorylation of 23 positions (Supplementary Table 2). Combining Myc-immunostaining and phosphorylation sites together with the bioinformatics analysis allowed us to construct a model topology, which predicts 38 transmembrane domains with intracellular N- and C-termini (Fig. 1b). However, this might be an over-estimation of transmembrane domains, since the prediction heavily relies on bioinformatics, and because we were unable to get experimental evidence for many of the predicted extracellular loops. If we instead consider that Myc tags detected only after permeabilization may be indicative of intracellular or transmembrane positions, then a second model with less TM domains can be proposed (Fig. 1c). In this model, among the 35 Myc tags detected only after permeabilization, only one is predicted to be extracellular. Indeed, Myc 1126 is expected to be present in the extracellular loop labelled by Myc 1071 in both models because no predicted TM domain or hydrophobic domain is present between Myc 1071 and Myc 1126. Therefore, absence of Myc 1126 staining without permeabilization is likely due to accessibility issue. In this second model, mPiezo1 is predicted to have a minimum of 18 TM domains. In addition, if we further assume that the inter-hydrophobic loops with phosphorylated residues are the only four intracellular loops, then only 10 of the hydrophobic regions would be counted as transmembrane domains. This extreme scenario would predict a topology with a minimum of 10 TM domains.

### Pore-related properties of mPiezo1/dPiezo chimeras

**Figure 2:**
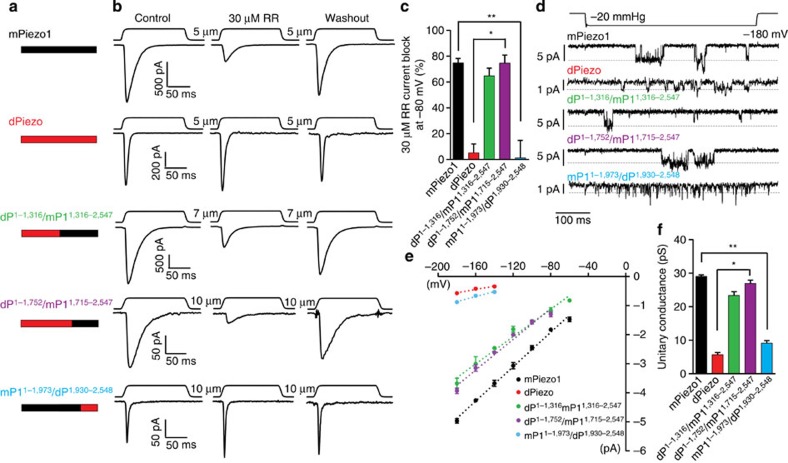
Ruthenium red sensitivity and single-channel properties of mPiezo1 and dPiezo chimeras. (**a**) Schematic representations illustrating portions of the protein sequence from mPiezo1 (black) or dPiezo (red) for each construct. (**b**) Representative (from five to eight experimental replicates) MA current traces at −80 mV from cells transfected with constructs shown in **a** before, during or after application of 30 μM RR. Each trace is an average of —three to five trials. Probe stimulation displacements are indicated. (**c**) Percent inhibition of MA currents in cells transfected with specified constructs in the presence of 30 μM RR (*n*=8, 5, 6, 5 and 5; mean±s.e.m.). One-way analysis of variance (ANOVA) with Dunn’s comparison with mPiezo1 or dPiezo, *P<0.05, **P<0.01. (**d**) Representative (from five to seven experimental replicates) stretch-activated channel openings elicited at −180 mV from cells transfected with specified constructs. dPiezo and mP1^1–1,973^/dP^1,930–2,548^ traces are displayed after applying 1 kHz digital filter. (**e**) Average I–V relationships of stretch-activated single channels in cells transfected with specified constructs. Single-channel amplitude was determined as the amplitude difference in Gaussian fits of full-trace histograms. (**f**) Unitary conductance of stretch-activated channels from cells transfected with specified constructs. Conductance is calculated from the slope of linear regression line of individual cell single-channel I–V relationships (*n*=7, 5, 5, 5 and 6; mean±s.e.m.). One-way ANOVA with Dunn’s comparison with mPiezo1 or dPiezo, *P<0.05, **P<0.01.

We next set out to identify residues involved in the ion-permeation pathway of the channel. mPiezo1 and dPiezo differ in their biophysical pore properties, such as sensitivity to the polycationic pore blocker ruthenium red (RR) and single-channel conductance^14^. We therefore generated chimeras between mPiezo1 and dPiezo based on our predicted mPiezo1 topology, and assayed them for these pore-related properties (Fig. 2). All chimeras were expressed in HEK293T cells and tested in whole-cell configuration to determine RR sensitivity of MA currents, and in cell-attached configuration to determine unitary conductance of stretch-activated channels.

Replacing the C-terminal half of mPiezo1 amino-acid sequence with dPiezo (chimera mP1^1–1,315^/dP^1,317–2,548^) generated a non-functional channel but the reciprocal substitution (chimera dP^1–1,316^/mP1^1,316–2,547^) gave rise to a chimeric channel with MA currents sensitive to 30 μM RR, similar to mPiezo1 but not to dPiezo (Fig. 2a–c and Supplementary Table 3). Single-channel conductance of this chimeric channel was also similar to mPiezo1 (Fig. 2d–f and Supplementary Table 3), demonstrating that biophysical pore properties of Piezo channels are determined by the C-terminal half of the protein sequence. We subsequently generated more chimeras by swapping shorter C-terminal regions between the two proteins. Chimera mP1^1–1,714^/dP^1,753–2,548^ was not functional, and chimera dP^1–1,929^/mP1^1,974–2,547^ could not be generated, but the complementary chimeras, dP^1–1,752^/mP1^1,715–2,547^ and mP1^1–1,973^/dP^1,930–2,548^, were functional and analysed. Chimera dP^1–1,752^/mP1^1,715–2,547^ displayed RR sensitivity and unitary conductance similar to mPiezo1, whereas chimera mP1^1–1,973^/dP^1,930–2,548^ had channel properties comparable to dPiezo (Fig. 2 and Supplementary Table 3). These results suggest that domain(s) required for determining conductance and pore block are encoded by the region of the protein sequence starting from mPiezo1 amino acids 1974. Interestingly, this segment of dPiezo and mPiezo1 sequences are 40% identical compared with 24% identity for the entire sequences. We also observed that whole-cell inactivation properties were likely dependent on this C-terminal region, as chimera dP^1–1,316^/mP1^1,316–2,547^ and dP^1–1,752^/mP1^1,715–2,547^ display inactivation kinetics comparable to mPiezo1, whereas mP1^1–1,973^/dP^1,930–2,548^ displays faster inactivation similar to dPiezo (Supplementary Table 3).

### Neutralization of acidic residues in the C-terminal region

**Figure 3:**
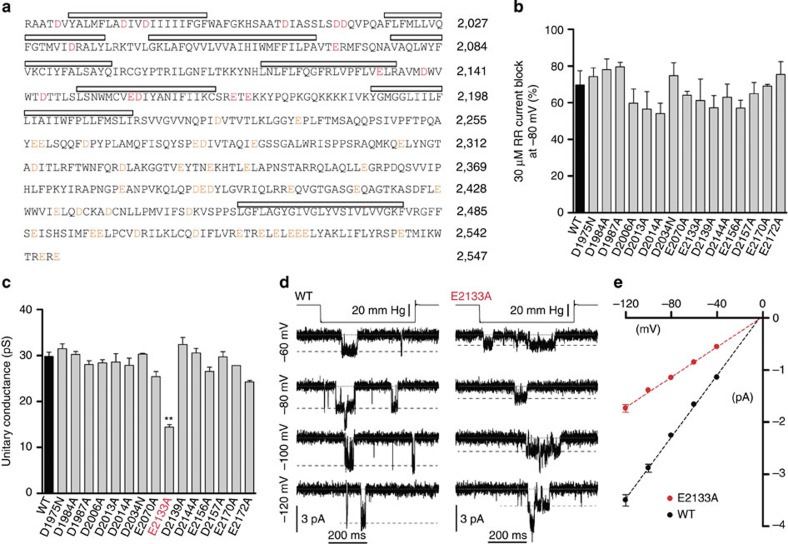
Neutralization of mPiezo1 acidic residues. (**a**) Protein sequence of mPiezo1 C-terminal region beginning at residue 1,971. Mutated acidic residues and other acidic residues are highlighted (red and orange, respectively). Grey bars indicate hydrophobic regions. (**b**) Average block of MA currents at −80 mV by 30 μM RR in cells transfected with mPiezo1 WT and specified mutants (*n*=2–6, mean±s.e.m.). (**c**) Unitary conductance of stretch-activated channels in cells transfected with WT mPiezo1 and specified mutants. Conductance is calculated from the slope of linear regression line of individual cell single-channel I–V relationships (*n*=3–13, mean±s.e.m.; One-way analysis of variance (ANOVA) with Dunn’s comparison to WT, **P<0.01). (**d**) Representative (from 13 and 5 experimental replicates) stretch-activated channel openings elicited at specified potentials from mPiezo1 WT and E2133A transfected cells. (**e**) Average I–V relationships of stretch-activated single channels in mPiezo1 WT and E2133A transfected cells (*n*=13 and 5, respectively; mean±s.e.m.). Single-channel amplitude was determined as the amplitude difference in Gaussian fits of full-trace histograms.

We performed site-directed mutagenesis to identify the molecular determinants that control ion-permeation properties of Piezo channels. Single point mutations in pore-forming domains of channels alter ion selectivity, single-channel conductance and pore block, and this approach has been widely used to prove that a protein is truly a pore-forming subunit^18^,^19^,^20^,^21^,^22^,^23^,^24^. Studies of classical Na^+^ and Ca^2+^ channels have shown that divalent/monovalent selectivity depends on charge density in the selectivity filter, the narrowest region that lines the pore. For example, the high calcium selectivity of Ca^2+^ channels depends on the high negative charge density in their selectivity filter^18^. Similarly, TRPV1–4 channels, which are non-selective cation channels with a P_Ca_/P_Na_ ratio in the 1–10 range^25^, contain a single acidic residue in their selectivity filter^26^. The neutralization of this acidic residue in TRPV1 and TRPV4 reduces the channel permeability for Ca^2+^and its affinity for the pore blocker RR^27^,^28^. Since mPiezo1 channels are non-selective cation channels^13^, we hypothesized that acidic residues may be a component of the putative selectivity filter controlling ion-permeation properties in mPiezo1. To this end, based on the data from our last chimeric channel (mP1^1–1,973^/dP^1,930–2,548^), we performed an alanine scan of acidic residues located in inter- or intra- hydrophobic domains encompassed by residues 1,974–2,547. Since there are no acidic residues within or adjacent to the last two predicted TM domains, we restricted our scan to the 15 aspartate and glutamate residues of mPiezo1 1,974–2,188 region (Fig. 3a). Of note, only 4 of these 15 acidic residues are not conserved in dPiezo; one of them (D2013) is replaced by another acidic residue (E), and the remaining three (D1987, D2014 and E2170) by neutral residues (N, N and Q, respectively).

Of the 15 individual alanine mutants, D1975A and D2034A were non-functional mutants, but the neutralization of aspartate residues with asparagine substitution resulted in functional clones. Therefore, we used D1975N and D2034N for further characterization. None of the mutations significantly affected the channel’s sensitivity to 30 μM RR (60 to 79% block at −80 mV) (Fig. 3b and Supplementary Table 3). Remarkably, one of the 15 mutations, E2133A, caused a strong reduction of single-channel current amplitude (Fig. 3c–e and Supplementary Table 3). The unitary conductance of E2133A mutant is half the unitary conductance of WT channels (14.4±0.5 and 29.1±0.4 pS, respectively; mean±s.e.m.). This suggests that E2133 is located in or near the mPiezo1 ion-conducting pathway.

### Mutation of conserved residues surrounding E2133

**Figure 4:**
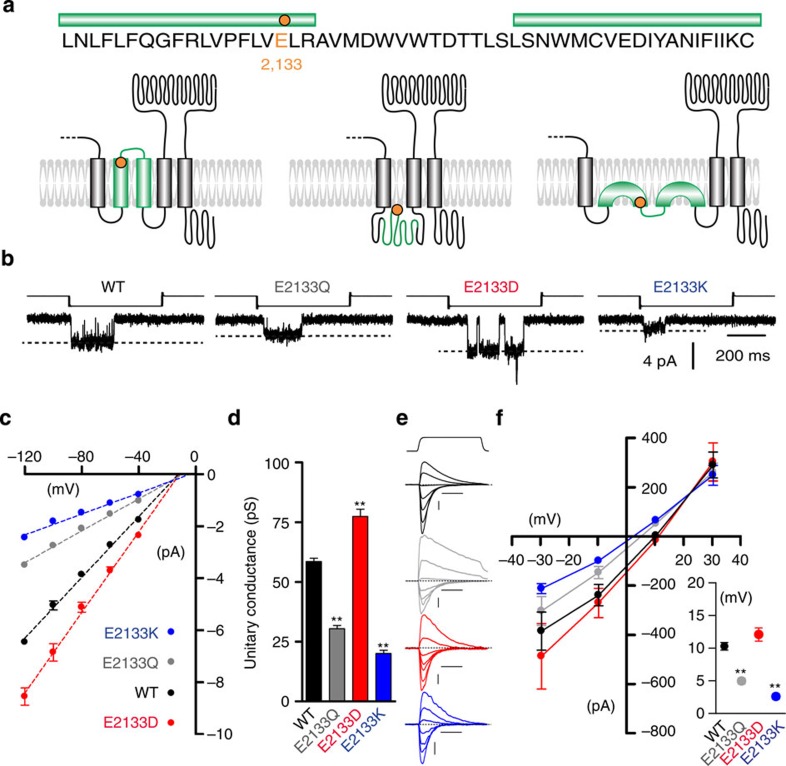
Mutations of a conserved glutamate residue alter mPiezo1 pore properties. (**a**) Protein sequence surrounding E2133 with putative topology models of mPiezo1 C-ter region. E2133 is highlighted (orange) and green bars indicate hydrophobic regions. (**b**) Representative (from four to seven experimental replicates) stretch-activated channel openings at −80 mV from cells transfected with mPiezo1 WT, E2133Q, E2133D and E2133K. Stimulation intensities are −15, −10, −20 and −50 mm Hg, respectively. (**c**) Average I–V relationships of stretch-activated single channels in cells transfected with mPiezo1 WT, E2133Q, E2133D and E2133K (*n*=7, 5, 5 and 4, respectively; mean±s.e.m.). Single-channel amplitude was determined as the amplitude difference in Gaussian fits of full-trace histograms. (**d**) Unitary conductance calculated from the slope of linear regression line of individual cells (mean±s.e.m.; One-way analysis of variance (ANOVA) with Dunn’s comparison to WT, **P<0.01). (**e**) Representative (from six to nine experimental replicates) traces of MA currents recorded with 150 mM CsCl-based intracellular solution and 100 mM CaCl_2_ extracellular solution from mPiezo1 WT (black), E2133Q (grey), E2133D (red) and E2133K (blue) transfected cells. Currents are elicited from −69.6 to +50.4 mV, Δ20 mV. Scale bars, 100 pA, 50 ms. Probe stimulation displacements are 3, 4, 6 and 7 μm, respectively. (**f**) Average I–V relationships of MA currents recorded from mPiezo1 WT, E2133Q, E2133D and E2133K expressing cells with 150 mM CsCl-based intracellular solution and 100 mM CaCl_2_ extracellular solution (*n*=8, 6, 6 and 9, respectively). Inset: average reversal potentials from individual cells corresponding to experiments in **f** (WT: 10.3±0.6 mV; E2133Q: 5.0±0.5 mV; E2133D: 12.1±1.0 mV and E2133K: 2.6±0.4 mV; mean±s.e.m.; One-way ANOVA with Dunn’s comparison with WT, **P<0.01). Experiments shown in **b**, **c** and **d** were performed in cell-attached configuration with Na^+^ as the only permeating cation in the recording pipette.

Alignment of Piezo protein sequences from various species highlights conservation of the region around mPiezo1 E2133 (Supplementary Fig. 4). We therefore tested if any of these conserved residues affect channel conductance. Removing Ca^2+^ from the recording solution abolishes Ca^2+^ block of these channels, resulting in a larger single-channel conductance and hence a higher signal-to-noise ratio. Indeed, mPiezo1 unitary conductance is doubled in Na^+^- or K^+^-based solution compared with standard pipette solution^14^. Under these conditions, E2133A unitary conductance is reduced by 2-fold compared with WT channels (26.8±0.6 and 58.6±1.2 pS, respectively) (Supplementary Fig. 5a–c and Supplementary Table 3). Individual alanine substitution of conserved residues upstream and downstream of E2133 resulted either in non-functional channels (P2129A, L2131A, R2135A and W2140A) or in channels with unitary conductance similar to WT channels (F2130A; 56.3±1.7 pS, V2132A; 59.5±1.7 pS and L2134A; 59.4±2.1 pS; mean±s.e.m.). In addition, we observed that in the absence of divalent ions, D2139A and D2144A mutations, which are downstream of E2133, result in slightly increased single-channel conductance compared with WT (64.2±1.2 and 69.6±1.8 pS, respectively; mean±s.e.m.). We next tested ion selectivity of these alanine mutants by measuring current–voltage relationship in 150 mM CsCl/100 mM CaCl_2_ asymmetric conditions (Supplementary Fig. 5d). Only E2133A mutation showed a significant shift in reversal potential relative to WT (Supplementary Fig. 5e). These results show that among the conserved residues in this region, E2133 is an important determinant of channel conductance and ion selectivity. mPiezo1 E2133 is surrounded by hydrophobic domains. On the basis of the predicted topologies presented in Fig. 1, this residue could be present in the membrane facing the extracellular side, in an intracellular loop, or in a structure analogous to a re-entrant loop (Fig. 4a).

### E2133 mutations influence mPiezo1 pore properties

**Figure 5:**
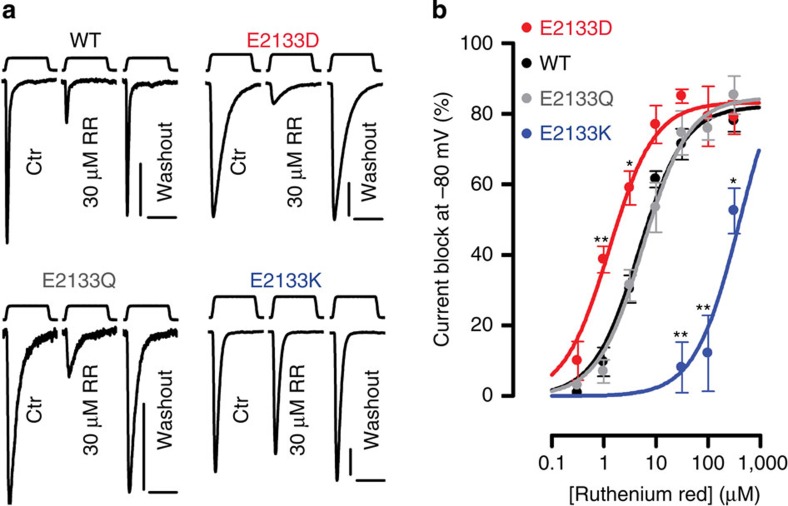
RR sensitivity of E2133 mutants. (**a**) Representative (from 3 to 10 experimental replicates) MA current traces from cells transfected with mPiezo1 WT, E2133Q, E2133D and E2133K at −80 mV before, during or after application of 30 μM RR. Each trace is an average of two to four trials. Probe stimulation displacements are 4.5, 5, 6 and 8 μm, respectively. Scale bars, 300 pA, 100 ms. (**b**) RR concentration–inhibition curves on MA currents at −80 mV in cells transfected with specified constructs (*n*=2–10 per data point; mean±s.e.m.). One-way analysis of variance (ANOVA) with Dunn’s comparison with WT done separately for each RR concentration, **P<0.01, *P<0.05.

We next tested whether changing the size/charge of the residue at position 2,133 modulates channel conductance, ion selectivity or RR sensitivity (Fig. 4 and Fig. 5). Three additional mutations were generated: E2133Q neutralizes the charge while keeping a similar side-chain length, E2133D keeps the negative charge but reduces the side-chain length, and E2133K replaces the negative charge with a positive one. In Na^+^-based pipette solution, all these substitutions significantly altered unitary conductance compared with WT channels. Unitary conductance of E2133Q was similar to E2133A mutants, whereas unitary conductance of E2133D and E2133K increased or drastically decreased compared with WT (77.5±3.0 and 20.0±1.3 pS, respectively; mean±s.e.m.; Fig. 4b–d and Supplementary Table 3). To determine whether these mutations affected ion selectivity of the channel, recordings were again made in 150 mM CsCl/100 mM CaCl_2_ asymmetric conditions. Under these conditions, E2133Q and E2133K mutations significantly shift the channel’s reversal potential to the left (5.0±0.5 and 2.6±0.4 mV, respectively, versus 10.3±0.6 mV for WT; mean±s.e.m.; Fig. 4e,f). These results show that E2133 is a determinant of ion selectivity, and the shift in reversal potential could be caused either due to a decrease in calcium permeability or due to an increase in chloride permeability. Therefore, we explored these two possibilities by determining chloride and calcium permeability for E2133Q and E2133K mutants. We found that mPiezo1 permeates chloride with P_Cl_/P_Na_ of 0.14, and that E2133K mutation significantly increases this ratio by 1.5-fold compared with WT (Supplementary Fig. 6a,b). This shows that introducing a positive charge at position 2,133 decreases mPiezo1 preference for cations over anions. On the contrary, chloride permeability of E2133Q mutant was similar to WT and therefore cannot explain the difference in reversal potential observed in the 150 mM CsCl/100 mM CaCl_2_ condition, suggesting that Ca^2+^ permeability was altered in this mutant. Indeed, we found a P_Ca_/P_Cs_ ratio of 1.21 for mPiezo1 which is decreased by 1.3-fold in E2133Q mutant (Supplementary Fig. 6c,d). These results highlight the importance of the charge present at position 2,133 for mPiezo1 conductance and ion selectivity.

RR is a voltage-dependent extracellular pore blocker of mPiezo1 (ref. 14). Mutation of acidic residues in the selectivity filter of TRPV channels affects sensitivity to RR^26^,^27^,^28^. Therefore, we tested whether RR sensitivity was modulated by E2133Q, D and K substitutions (Fig. 5). At −80 mV, 30 μM RR blocked MA currents from E2133Q and E2133D channels by 74.6±6.2 and 85±2 % respectively, compared with 71.4±4.4 % for WT channels (mean±s.e.m.). E2133K, on the other hand, was only slightly blocked (8.1±7.1 %) by 30 μM RR. RR dose–response curves measured at −80 mV gave IC_50_ values of 5.62±1.27 μM and 1.26±0.28 μM for E2133Q and E2133D, respectively, compared with 4.71±0.83 μM for WT (mean±s.e.m.) , illustrating that RR sensitivity of E2133Q is similar to WT, whereas E2133D channels are slightly more sensitive. Remarkably, RR IC_50_ for E2133K channels was about 80-fold higher than WT (Fig. 5b). Neutralization of the E2133 residue did not relieve RR block; therefore, the glutamate is unlikely to be the RR binding site. The positively charged lysine substitution, however, could alter the local charge in the vicinity of the putative RR binding site, thereby altering the electrostatic interaction between RR and the channel^29^. Additional studies in this region will determine the molecular mechanism of RR binding and inhibition in Piezos.

To test whether the E2133K substitution, which had the most prominent consequence on pore properties, could also alter pressure sensitivity of the channel, we compared the current–pressure relationships of WT and E2133K channels in cell-attached mode. We did not detect a change in pressure sensitivity, suggesting that although E2133K affects pore-permeation properties, it does not alter the channel’s mechanical sensitivity (Supplementary Fig. 7).

### Conserved glutamate determines Piezo2 pore properties

**Figure 6:**
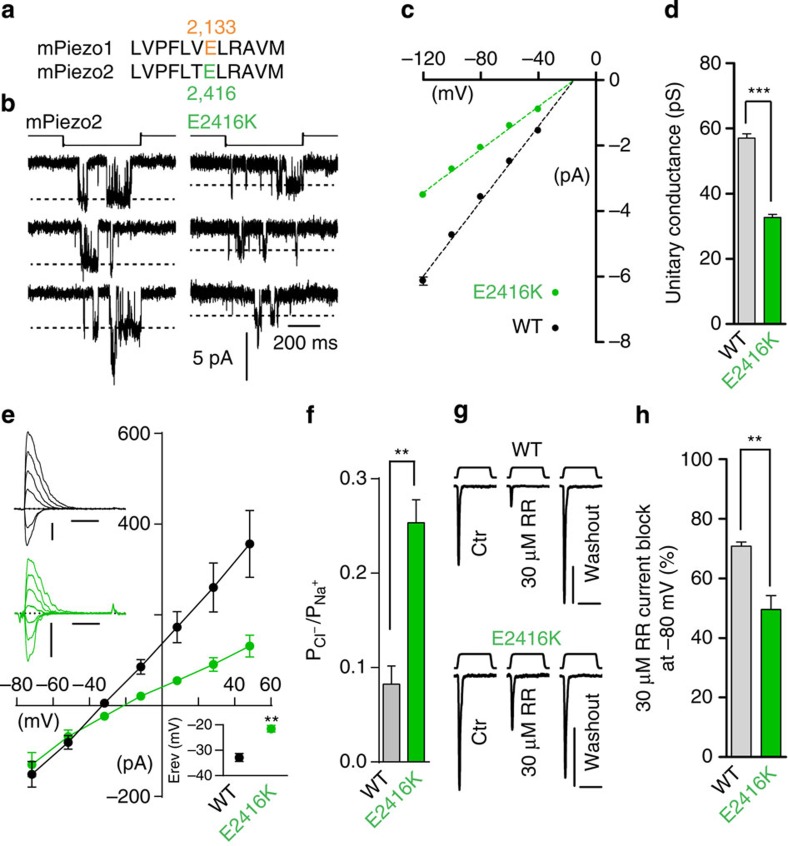
Lysine substitution of E2416 alters pore properties of mPiezo2 channels. (**a**) Conservation of mPiezo1 E2133 at position 2,416 on mPiezo2 protein. (**b**) Representative (from nine and six experimental replicates) stretch-activated channel openings at −80 mV from cells transfected with mPiezo2 WT and E2416K. Stimulation intensities are −15 and −20 mm Hg, respectively. (**c**) Average I–V relationships of stretch-activated single channels in mPiezo2 WT and E2416K transfected cells (*n*=9 and 6, respectively; mean±s.e.m.). Single-channel amplitude was determined as the amplitude difference in Gaussian fits of full-trace histograms. (**d**) Single-channel conductance calculated from the slope of linear regression line of individual cell single-channel I–V relationships (mean±s.e.m.; Mann–Whitney test, ***P<0.001). (**e**) Average I–V relationships of MA currents recorded from mPiezo2 WT and E2416K expressing cells with 150 mM NaCl-based intracellular solution and 30 mM NaCl extracellular solution. Top left inset: typical recording traces for WT (black) and E2416K (green) from −71.7 to +48.3 mV, Δ20 mV. Scale bars, 100 pA, 50 ms. Probe stimulation displacements are 9 and 8 μm, respectively. Bottom right inset: average reversal potential (mean±s.e.m.; *n*=6 and 5, respectively; Mann–Whitney test **P<0.01). (**f**) P_Cl_/P_Na_ permeability ratios of MA currents from cell transfected with mPiezo2 WT, and E2416K (mean±s.e.m.; *n*=6 and 5, respectively; Mann–Whitney test **P<0.01). (**g**) Representative (from five experimental replicates) MA current traces from mPiezo2 WT and E2416K transfected cells at −80 mV before, during or after application of 30 μM RR. Each trace is an average of two to four trials. Probe stimulation displacements are 5 and 7 μm, respectively. Scale bars, 100 pA, 100 ms. (**h**) Average block of MA currents at −80 mV by 30 μM RR in mPiezo2 WT and E2416K transfected cells (*n*=5 for each; mean±s.e.m.; Mann–Whitney test, **P<0.01). Experiments shown in **b**, **c** and **d** were performed in cell-attached configuration with Na^+^ as the only permeating cation in the recording pipette.

We next tested whether the conserved glutamate residue is also functionally required to determine pore properties in another Piezo ion channel by mutating the homologous residue in mPiezo2, E2416, into a Lysine (Fig. 6a). We focused on mouse Piezo2 instead of Drosophila Piezo since the fly channel is not blocked by RR, and its small single-channel currents are challenging to record (a 2-fold decrease in conductance would be difficult to validate). We recorded stretch-activated single-channel currents from HEK293T cells expressing mPiezo2 and found the unitary conductance in Na^+^-based pipette solution condition to be 56.9±1.3 pS. Under these conditions, the unitary conductance of mPiezo2 E2416K mutant was reduced by 1.7-fold relative to WT (32.6±1.0 pS; mean±s.e.m.; Fig. 6b–d and Supplementary Table 3). In addition, E2416K mutation also alters ion selectivity of the channel (Supplementary Fig. 8), decreasing cation over anion preference illustrated by a 3-fold increase of P_Cl_/P_Na_ ratio (0.08 and 0.25 for WT and E2416K, respectively; Fig. 6e,f). We next tested the extent of RR block of MA whole-cell currents from WT and E2416K mPiezo2 expressing cells. Compared with WT, E2416K mutant channels were significantly less sensitive to 30 μM RR (70.8±1.4 and 49.6±4.6 % blocks, respectively; mean±s.e.m.; Fig. 6g,h and Supplementary Table 3). Therefore, similar to mPiezo1, lysine substitution of E2416 in mPiezo2 alters conductance, ion selectivity and pore block of the channel. These results suggest a general requirement for this conserved glutamate residue in controlling pore properties and indicates that the hydrophobic regions adjacent to the last two predicted TM domains of the channel could be either part of the pore domain or closely associated with the pore across Piezo channels.

## Discussion

In this study, we describe the first structural motif of Piezo channel family. We combined bioinformatics, intramolecular Myc-tag and phosphorylated site detection to begin to unlock the transmembrane topology of mPiezo1 protein. By developing a chimeric approach between mouse Piezo1 and drosophila Piezo, we show that the portion of mPiezo1 from 1,974 to C terminus is essential for ion-permeation properties. Further, we show that a conserved glutamate residue within hydrophobic regions neighbouring the last two putative TM domains alter pore-dependent properties, suggesting that this residue is either within or in close proximity to the mPiezo1 pore-forming domain.

We provide the first experimental evidence validating certain regions of bioinformatically predicted mPiezo1 topology. We show that mPiezo1 has intracellular N- and C-terminals, and verify the orientation of 13 inter-hydrophobic domain loops of the protein. Piezo proteins are encoded by more than 2,000 residues and lack homology with any other protein^13^. The large size of the protein has proved challenging in delineating the exact topology of this non-selective cation channel. Several bioinformatics predictions estimate a topology consisting of anywhere between 30 and 40 TMs domains^11^,^16^,^30^,^31^,^32^,^33^,^34^,^35^,^36^. We experimentally addressed the transmembrane topology of Piezo proteins by generating 48 Myc insertion mutations in mPiezo1-predicted loops. Immuno-detection of these tags in non-permeabilized conditions was used to probe extracellular loops. We combined this approach with the detection of 23 phosphorylation sites by mass spectrometry to identify intracellular loops. Our results verify that nine of these putative loops have an extracellular orientation, and that along with the N- and C- terminal of the protein, four loops are intracellularly located. Although this advances our knowledge on topology, we cannot predict the exact number of transmembrane domains. On the basis of this data, a model that predicts 38 TM domains best agrees with the bioinformatics prediction of mPiezo1 topology. However, we do not have experimental data proving that many of the loops that are proposed to be present on the extracellular side in this model are indeed accessible from outside the cell. This is not completely unexpected, since Myc tags inserted in small extracellular loops might not be accessible to the Myc antibody in live cells. But it is also possible that the lack of labelling could point to these loops being intracellular. Catering to this possibility, if we assume that all Myc tags not observed in live unpermeabilized cells are intracellular or within transmembrane regions, then mPiezo1 could have only 18 TM domains. Furthermore, if we assume that only regions that are phosphorylated form intracellular loops, then a topology with a minimum of 10 putative TM domains could be proposed. The actual number of TM domains could be anywhere in between the two extreme cases. Regardless, our results here represent the first experimentally based information on Piezo topology, and will be invaluable for future studies addressing structural features of this channel.

We generated chimeric proteins between mPiezo1 and dPiezo and assayed their pore-related properties^14^. We show that the mPiezo1 C-terminal domains encoded by the residues 1,974–2,547 confer unitary conductance, ion selectivity and RR sensitivity. Therefore, we conclude that C-terminal portion of mPiezo1, which is 1/5th of the whole protein, includes most if not all of the pore domain. Interestingly, according to our predicted mPiezo1 topology, ∼70% of the reported hPiezo1 and hPiezo2 mutations map to this region, including hPiezo1 (T2127M) and hPiezo2 (T2356M), which are in close proximity to our identified glutamate residue (see below)^16^, emphasizing the importance of this region in channel function.

Following up on our chimeric channel screen, we used site-directed mutagenesis of acidic residues within hydrophobic regions encoded by residues 1,974–2,188 to show that neutralization of a single glutamate residue results in a 2-fold decrease in unitary conductance. Additional substitutions of aspartate, lysine or glutamine instead of the glutamate residue altered unitary conductance, ion selectivity and sensitivity to RR, thereby suggesting that E2133 contributes to mPiezo1 pore properties. The role of this glutamate residue in dictating ion-permeation properties of Piezos was further confirmed when the lysine substitution of the homologous residue in mPiezo2 (E2416K) also modulated unitary conductance, cation over anion selectivity and RR sensitivity.

Alignment of Piezo proteins from distant species such as parasites shows that mPiezo1 E2133 glutamate residue is the only charged residue in a conserved stretch of amino acids that form a PF(X_2_)E(X_6_)W motif^34^. We found that P and W residues are indeed essential for channel function, as mutations at these positions result in non-functional channels. The impact of the glutamate residue on pore properties and its location in a highly conserved motif is suggestive of its presence in a structure similar to a selectivity filter present in most members of known ion channel families. However, only lysine substitution of the E2133 altered mPiezo1 RR sensitivity, suggesting that this residue is not the RR binding site. Furthermore, the magnitude of the impact that lysine substitution has on ion selectivity and single-channel conductance is rather mild for a model where the selectivity filter of mPiezo1 or 2 channels would constitute a ring of E2133 or E2416 residues from associated subunits. This suggests that the glutamate residue may not lie in the selectivity filter but could be located close enough to the pore to allosterically modulate its properties.

The E2133 residue lies in a region flanked by hydrophobic residues. However, the different topology models differ in the prediction of how these hydrophobic domains are oriented. It is possible that the hydrophobic domains form full transmembrane segments, and that E2133 could be accessible from or close to the extracellular side (Fig. 1b). Alternatively, it might be located closer to the intracellular side, potentially as part of a re-entrant loop (Fig. 1c). The true orientation of this E2133 region relative to the rest of the channel will be elucidated by future structural analysis.

Although mutating the glutamate residue impacts pore properties in mouse Piezos, this residue is conserved between mouse (E2133/2416 in mPiezo1/2) and fly Piezos (E2091 in dPiezo) and therefore cannot explain their pore-related differences. Therefore, other unidentified differences between mouse and fly Piezos in the C-terminal region must explain the large variations in their biophysical pore properties.

Overall, we provide strong evidence that the protein region corresponding to mPiezo1 residues 1,974 to C terminus encompass the domains that form the pore of the channel, and that mPiezo1 E2133 residue (or mPiezo2 E2416) is at least in the vicinity of the pore-permeation pathway and can influence Piezo pore properties.

## Methods

### mPiezo1 topology prediction

Using the sequence of mammalian Piezos, topology predictions were made using Hopp–Woods hydrophilicity/antigenicity plots as well as Kyte Doolittle hydrophobicity plots. A combination of these results was used to predict the final topology. Our approach was validated to predict the topology of various TRP channels and CLC channels^37^. For Hopp–Woods plots, we used a window size of 7 to predict highly hydrophilic/antigenic regions. For Kyte Doolittle hydrophobicity plots, we used a window size of 17 to predict TM regions and a window size of 9 to predict surface regions over the full-length amino-acid sequence. For ambiguous regions, fragments of the amino-acid sequence were analysed separately using the same parameters. The results were then compared with putative transmembrane domains predicted by TMHMM2 and Phobius algorithms. A total of 38 transmembrane regions were predicted. Myc tags were inserted in predicted inside and outside loops near hydrophilic residues, preferably in the middle of the loop. All Myc constructs were generated using the QuickChange II XL site-directed mutagenesis kit (Agilent Technologies). Myc tags were also introduced at the N- and C-terminal regions. All constructs were verified by sequencing.

HEK293T cells were plated on poly-D-lysine-coated MatTek dishes, which were additionally coated with laminin (10 or 20 μg ml^−1^). Cells were transfected with each construct separately (2 μg cDNA each) using Fugene HD (Promega). Twenty-four to 48 h after transfection live labelling was carried out by incubating transfected HEK293T cells with Myc 9E11 (1:50; Santa Cruz Biotechnology) at 37 °C for 20 min or 1 h. After five washes with warm medium, cells were incubated with secondary antibodies conjugated to Alexa Fluor 546 (1:200; Life Technologies) for 10–20 min at room temperature. Cells were washed five times with PBS, fixed with 2% PFA/PBS for 20–30 min and imaged at an Olympus (Tokyo, Japan) Fluoview 500 confocal microscope by illumination with the HeNe green 543 nm laser.

Immunostaining after fixation and permeabilization of cells was carried out essentially as described^13^. In brief, cells were fixed with 4% PFA/PBS for 10 min, washed five times with PBS, permeabilized in PBS containing 0.4% Triton X-100 and blocked with 10% normal goat serum in PBS followed by incubation with antibodies. The Myc 9E11 (1:100; Santa Cruz Biotechnology) antibody was used to confirm predicted outside and inside loops and Piezo1 antibodies (1:100) to confirm Piezo1 expression. After three washes in PBS, secondary antibodies conjugated to Alexa Fluor 546 and 633 (1:200; Life Technologies) were applied. Immunostainings were imaged at an Olympus (Tokyo, Japan) Fluoview 500 confocal microscope by sequential illumination using the HeNe green 543 nm laser and the HeNe red 633 nm laser. The live labelling and permeabilized staining was repeated two or more times for each construct to confirm results.

### Detection of phosphorylation sites

mPiezo1-GST proteins were purified from HEK293T cells transfected with mPiezo1-GST cDNAs^14^. After incubation with cell lysates overnight at 4 °C, the glutathione beads were washed four times in a buffer containing 25 mM NaPIPES, 140 mM NaCl, 0.6% CHAPS, 0.14% phosphatidylcholine (PC), 2.5 mM dithiothreitol (DTT) and a cocktail of protease inhibitors and eluted with 100 mM glutathione in a buffer containing 25 mM NaPIPES, 50 mMTris, 0.6% CHAPS, 0.14% PC, 2.5 mM DTT and a cocktail of protease inhibitors. The eluant was dialysed against a buffer containing 25 mM NaPIPES, 0.6% CHAPS, 0.14% PC, 2.5 mM DTT and a cocktail of protease inhibitors. The protein samples were denatured, reduced and alkylated with 2-iodoacetamide before precipitation with acetonitrile. The pellet was re-dissolved in 8 M urea and ProteaseMax detergent, then diluted to 1 M urea and digested with trypsin or chymotrypsin. A portion of each digest was analysed by nanoscale liquid chromatography-mass spectrometry (nanoLC/MS) on the Orbitrap Velos (Thermo Scientific) without further processing. The remainder of the digests was enriched for phosphopeptides using TiO2 beads and also analysed by nanoLC/MS on the Orbitrap Velos. The data were searched using a small database (∼450 proteins) to identify peptides from mPiezo1 (∼73% sequence coverage) and sites of phosphorylation and the search results combined in Scaffold 3. The search results were filtered for high confidence (95% at the peptide level) and MS/MS indicating sites of phosphorylation were inspected.

### Generation of chimeras and mutants

mPiezo1 and dPiezo chimeras were generated by swapping specific regions in the C-terminal end of the protein. The beginning of a swapped region was chosen at a stretch of amino acids that were conserved between mPiezo1 and dPiezo. These junction sites are marked in Supplementary Fig. 2. Domain swapping was carried out using the QuickChange II XL site-directed mutagenesis kit from Agilent Technologies. Mega-primers were designed flanking the region of interest with overlapping base pairs between the donor and the recipient cDNA. The mega-primer was generated by PCR amplification and gel purification. The PCR product was then used as a primer in the mutagenesis reaction with the recipient cDNA as the template. PCR cycling conditions were according to the manufacturer’s instructions. Reactions were transformed into XL-gold competent cells and colonies screened. All positive chimera clones were verified by full-length DNA sequencing.

Point mutants were also generated using the QuickChange II XL site-directed mutagenesis kit according to the manufacturer’s instructions and confirmed by full-length DNA sequencing. dPiezo chimeras were cloned into pIres2-EGFP vector and all mPiezo1 chimeras and mutants were cloned into pcDNA3.1(−) vector, which was modified to include an IRES-EGFP element. mPiezo2 (E2416K) was cloned in pCMV-Sport6 vector.

### Cell culture and transient transfection

Human embryonic kidney 293T (HEK293T) cells were grown in Dulbecco's Modified Eagle Medium containing 4.5 mg ml^−1^ glucose, 10% fetal bovine serum, 50 units ml^−1^ penicillin and 50 μg ml^−1^ streptomycin. Cells were plated onto 12-mm round glass poly-D-lysine coated coverslips placed in 24-well plates and transfected using lipofectamine 2000 (Invitrogen) according to the manufacturer’s instruction. All plasmids were transfected at a concentration of 600 to 1,000 ng ml^−1^. mPiezo2(E2416K) was co-transfected with GFP at a concentration of 300 ng ml^−1^. Cells were recorded from 12 to 48 h post transfection.

### Electrophysiology

Patch-clamp experiments were performed in standard whole-cell or cell-attached mode using Axopatch 200B amplifier (Axon Instruments). Currents were sampled at 20 kHz and filtered at 2 kHz. Leak currents before mechanical stimulations were subtracted off-line from the current traces. Voltages were not corrected for a liquid junction potential except for ion selectivity experiments (Figs 4e–g and 5e,f, Supplementary Fig. S6D,E and S7). Liquid junction potential was calculated using Clampex 10.3 software. All the experiments were done at room temperature.

### Solutions

For whole-cell patch-clamp recordings, recording electrodes had a resistance of 2–3 MΩ when filled with internal solution composed of (in mM) 133 CsCl, 1 CaCl_2_, 1 MgCl_2_, 5 EGTA, 10 HEPES (pH 7.3 with CsOH), 4 MgATP and 0.4 Na_2_GTP. The extracellular solution was composed of (in mM) 133 NaCl, 3 KCl, 2.5 CaCl_2_, 1 MgCl_2_, 10 HEPES (pH 7.3 with NaOH) and 10 glucose. For ion-selectivity experiments, internal solution used was (in mM) 150 CsCl, 10 HEPES (pH 7.3 with CsOH) and extracellular solution consisted of (in mM) 100 CaCl_2_ and 10 HEPES (pH 7.3 with CsOH). P_Cl_/P_Na_ was measured in extracellular solution composed of (in mM) 30 NaCl, 10 HEPES and 225 Sucrose (pH 7.3 with NaOH) and intracellular solution consisted of (in mM) 150 NaCl and 10 HEPES (pH 7.3 with NaOH). P_Ca_/P_Cs_ was measured in extracellular solution composed of (in mM) 50 Ca-gluconate, 0.5 CaCl_2_, 10 HEPES and 170 sucrose (pH 7.3 with CsOH), and intracellular solution composed of (in mM) 149 Cs-methanesulfonate, 1 CsCl and 10 HEPES (pH 7.3 with CsOH). For ruthenium red (RR) experiments, 10 or 100 mM RR stock solution was prepared in water and diluted to working concentrations in extracellular solution.

For cell-attached patch-clamp recordings, external solution used to zero the membrane potential consisted of (in mM) 140 KCl, 1 MgCl_2_, 10 glucose and 10 HEPES (pH 7.3 with KOH). Recording pipettes were of 2–3 MΩ resistance when filled with standard solution composed of (in mM) 130 mM NaCl, 5 KCl, 1 CaCl_2_, 1 MgCl_2_, 10 TEA-Cl and 10 HEPES (pH 7.3 with NaOH) or divalent-free pipette solution consisted of (in mM) 150 mM NaCl, 10 HEPES (pH 7.3 with NaOH).

### Whole-cell mechanical stimulation

For whole-cell recordings, mechanical stimulation was achieved using a fire-polished glass pipette (tip diameter 3–4 μm) positioned at an angle of 80° relative to the cell being recorded. Downward displacement of the probe towards the cell was driven by Clampex-controlled piezoelectric crystal microstage (E625 LVPZT Controller/Amplifier; Physik Instrumente). The probe had a velocity of 1 μm ms^−1^ during the ramp phase of the command for forward movement and the stimulus was applied for 150 ms. To assess the mechanical sensitivity of a cell, a series of mechanical steps in 0.5 or 1 μm increments was applied every 10–20 s. For RR experiments, once an efficient stimulus eliciting MA current for a cell was achieved, repeated stimuli at that distance were then given every 20–30 s. Cells were continuously perfused and at least three consecutive stable recording traces were achieved before switching between solutions. Current parameters were measured from three or more averaged traces. For I–V relationship recordings, voltage steps were applied 700 ms before mechanical stimulation (150 ms) from a holding potential of −60 mV. Voltage steps were given from −80 mV to +80 in 20 mV increments. The starting position of the stimulation probe relative to the cell is not tightly controlled and varies between cells. Therefore, responses were recorded at different probe displacements from cell to cell allowing recording of MA currents with similar amplitude.

### Permeability ratio measurements

Reversal potential for each cell in the mentioned solution was determined by interpolation of the respective current–voltage data. Permeability ratios were calculated by using the following Goldman–Hodgkin–Katz (GHK) equations:

P_Cl_/P_Na_ ratios:




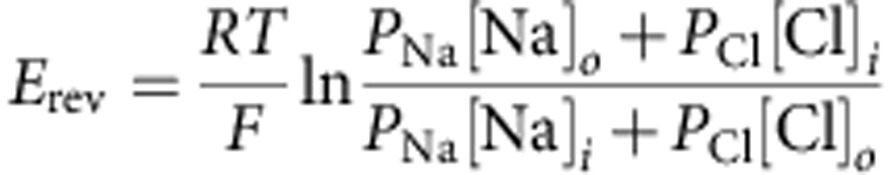




P_Ca_/P_Cs_ ratios:




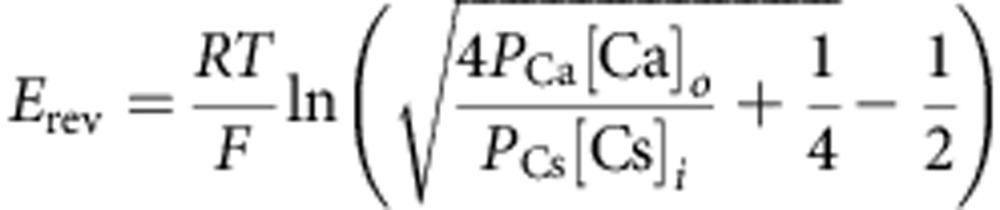




where, *RT*/*F* has the value 25.69 at 25 °C.

### Unitary conductance measurement

Stretch-activated currents were recorded in the cell-attached patch-clamp configuration. Membrane patches were stimulated with 500 ms negative pressure pulses through the recording electrode using Clampex-controlled pressure clamp HSPC-1 device (ALA-scientific). Since the single-channel amplitude is independent of the pressure intensity, the most optimal pressure stimulation was used to elicit responses that allowed single-channel amplitude measurements. These stimulation values were largely dependent on the number of channels in a given patch of the recording cell. Single-channel amplitude at a given potential was measured from trace histograms of 5 to 20 repeated recordings. Histograms were fitted with Gaussian equations using Clampfit 10.3 software or multi-peak fitting analysis of IGOR Pro software. Single-channel slope conductance for each individual cell was calculated from linear regression curve fit to single-channel I–V plots. We sometimes observed sub-conductance states in single-channel recording of chimeric and mutant channels, but we focused the analysis on the state that was prevalent.

Concerning mPiezo2 WT and mutant, stretch-activated currents were detected in only ∼50% of HEK293T transfected cells but never in cells expressing ires2-EGFP only. This frequency is lower than the one of mPiezo1 and related mutants (∼90% positive patches). It may be due to either lower expression of mPiezo2-related constructs or to unknown functional difference between mPiezo1 and 2. In standard pipette solution, mPiezo2 unitary conductance is 23.7±0.6 pS (Supplementary Table 3).

## Additional information

**How to cite this article:** Coste, B. *et al.* Piezo1 ion channel pore properties are dictated by C-terminal region. *Nat. Commun.* 6:7223 doi: 10.1038/ncomms8223 (2015).

## Supplementary Material

Supplementary Information Supplementary Figures 1-8, Supplementary Tables 1-3
