# Supplementary material for: Piezo1 ion channel pore properties are dictated by C-terminal region
Source: Nat Commun. 2015 May 26;6:7223. doi: 10.1038/ncomms8223 (PMC4445471; doi:10.1038/ncomms8223)
Supplement: Supplementary Information — Supplementary Figures 1-8, Supplementary Tables 1-3 [file ncomms8223-s1.pdf]

Piezo1 ion channel pore properties are dictated by C-terminal region

Bertrand Coste[1,2,5,*], Swetha E. Murthy[2,5], Jayanti Mathur[3], Manuela Schmidt[2,4], Yasmine Mechioukhi[1], Patrick Delmas[1], Ardem Patapoutian[2,3]

[1]Aix Marseille Université, CNRS, CRN2M-UMR7286, 13344 Marseille France.
[2]Howard Hughes Medical Institute, Molecular and Cellular Neuroscience, The Scripps Research Institute, La Jolla, California 92037, USA.
[3]Genomics Institute of the Novartis Research Foundation, San Diego, California 92121, USA.
[4]Present Address: Somatosensory Signaling Group; Max Planck Institute of Experimental Medicine, 37075 Goettingen, Germany.
[5]These authors contributed equally to this work
*Correspondence: bertrand.coste@univ-amu.fr (B.C.)

Supplemental Figures and Legends

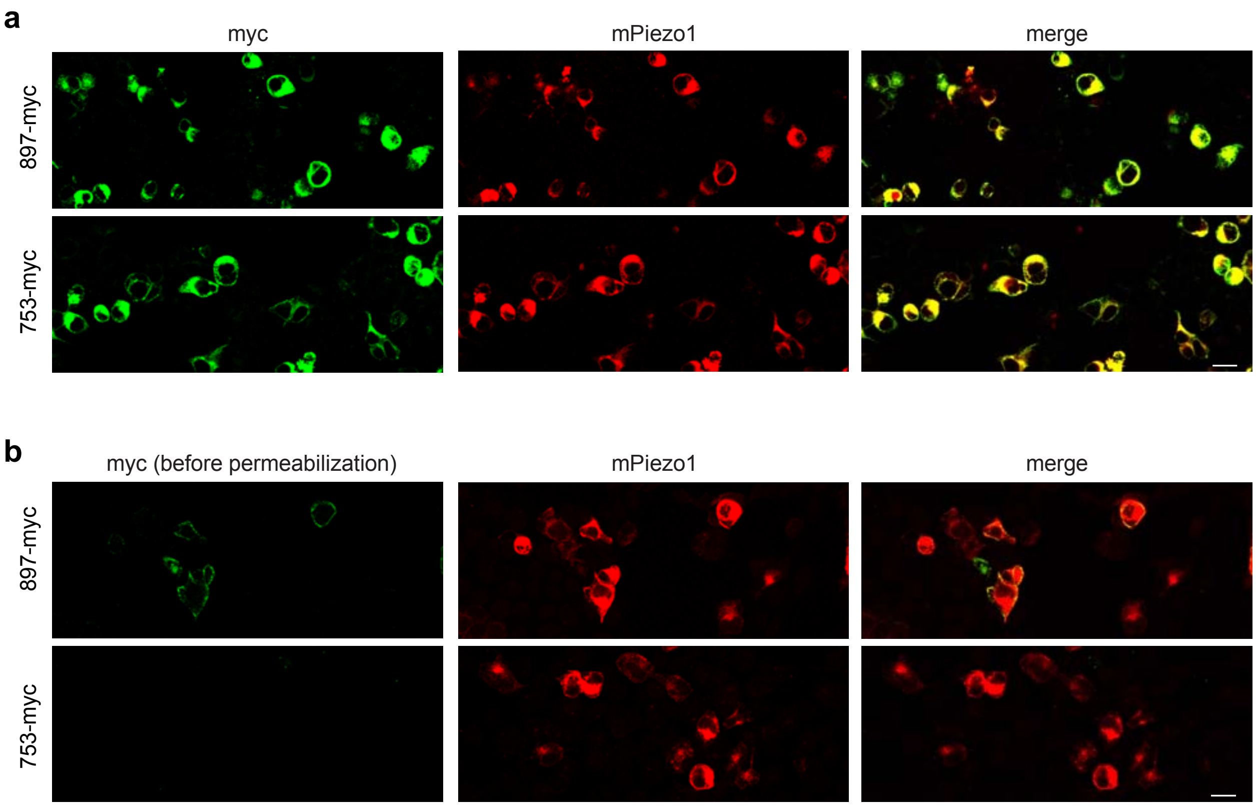

Supplementary Figure 1. Staining of mPiezo1-myc constructs using mPiezo1 antibody as a control of myc-tag localization experiments. (**a**) Representative images of labeling using a myc antibody (green, left panels) and mPiezo1 antibody (red, middle panels) in mPiezo1-myc transfected cells. Myc tags were inserted at position 897 or 753, as specified. Staining using myc- and mPiezo1-antibodies overlap (right panels), illustrating that the position of myc-tag insertion does not affect their accessibility. (**b**) Same as (**a**) except that myc staining was performed before permeabilization. The level of expression of both mPiezo1-myc constructs evaluated by mPiezo1 labelling is similar illustrating that lack of myc staining of 753-myc constructs under these conditions is not due to lower expression. Scale bar: 20 µm. Note that Myc labelling images in panel (**a**) and (**b**) are the same used in Fig1a, left and right panels, respectively.

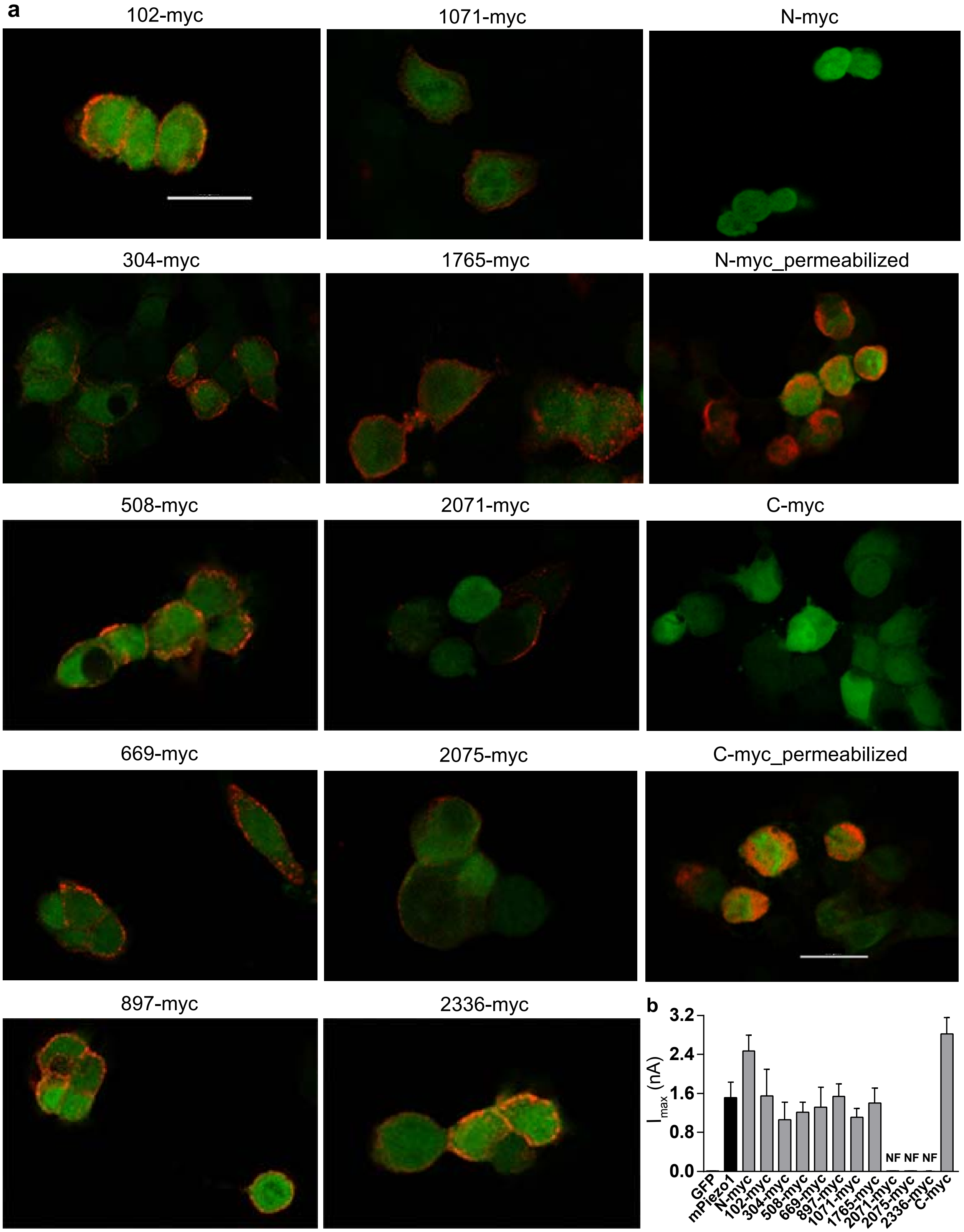
a
102-myc
1071-myc
N-myc
304-myc
1765-myc
N-myc_permeabilized
508-myc
2071-myc
C-myc
669-myc
2075-myc
C-myc_permeabilized
897-myc
2336-myc
b
3.2
2.4
1.6
0.8
0.0
I_{max} (nA)
NF NF NF
GFP
mPiezo1
N-myc
102-myc
304-myc
508-myc
669-myc
897-myc
1071-myc
1765-myc
2071-myc
2075-myc
2336-myc
C-myc

Supplementary Figure 2. Myc staining and functionality data of mPiezo1 myc-tag constructs. (**a**) Representative images of non-permeabilized and permeabilized staining using a myc antibody (red) in mPiezo1-myc iresGFP transfected cells. scale bar 20 µm. Note that extracellular myc detection signal of 2071- and 2075-myc constructs is weaker than for other constructs positively stained by anti-myc without permeabilization. This could reflect impaired membrane trafficking for these two constructs. (**b**) Bar graph representing MA whole-cell I_{max} currents recorded from cells transfected with GFP only, mPiezo1, or mPiezo1 with a myc tag at the following amino acid positions N-terminal, 102, 304, 508, 669, 897, 1071, 1765, 2071, 2075, 2336 and C-terminal (n= 9, 5, 4, 5, 6, 5, 5, 7, 7, 4, 5, 8, 9 and 5, respectively).

```
mPiezo1         MEPHVLGAGLYWLLLPCTLLAASLLRFNALSLVYLLFLLLLPWLPGPSRHSIPGHTGRLL 60
mPiezo2         MASEVVCGLIFRLLLPICLAVACAFRYNGLSFVYLIYLLLIPLFSEPTKATMQGHTGRLL 60
hPiezo1         MEPHVLGAVLYWLLLPCALLAACLLRFSGLSLVYLLFLLLLPWFPGPTRCGLQGHTGRLL 60
hPiezo2         MASEVVCGLIFRLLLPICLAVACAFRYNGLSFVYLIYLLLIPLFSEPTKTTMQGHTGRLL 60
dPiezo          MVFSYACMVLQRIVVPAVLVLAALMRPVGISFVYLLMFFVSPFVPLATRRNFKGSVTAFF 60

mPiezo1         RALLCLSLLFLVAHLAFQICLHTVPHLDQFL-GQNGSLWVKVSQHIGVTRLDLKDIFNTT 119
mPiezo2         QSLCITSLSFLLLHIIFHITLASLEAQHRITPAYNCSTWEKTFRQIGFESLKGADAGNGI 120
hPiezo1         RALLGLSLLFLVAHLALQICLHTVPRLDQLL-GPSCSRWETLSRHIGVTRLDLKDIPNAI 119
hPiezo2         KSLCFISLSFLLLHIIFHITLVSLEAQHRIAPGYNCSTWEKTFRQIGFESLKGADAGNGI 120
dPiezo          IILLTLSTLVLLGHITLQILAVSLT-----LPIYNCSFSERLLRHIGFVSFIDLQPFAII 115

mPiezo1         RLVAPDLGVLLASSLCLGLCGRLTRKAGQSRRTQELQDDDDDDDDDDEDIDAAPAVG--- 176
mPiezo2         RVFVPDIGMFIASLTIWLVCRTIVKKPDTEEIAQLNSECENEELAGGEKMDSEEALIYEE 180
hPiezo1         RLVAPDLGILVVSSVCLGICGRLARNTRQSPHPRELDDDE-------RDVDASPTAG--- 169
hPiezo2         RVFVPDIGMFIASLTIWLLCRNIVQKPVTDEAAQSNPEFENEELAEGEKIDSEEALIYEE 180
dPiezo          EWLVPEVLVFATSLGSYLTVKRVASQPVGAEQLENGEVVDGQAENAQTSSQPSAADANG- 174

mPiezo1         -----------LKGAPALATKRRLW-LASRFRVTAHWLLMTSGRTLVIVLLALA-----G 219
mPiezo2         DLDGEEGMEGELEESTKLKILRRFASVASKLKEFIGNMITTAGKVVVTILLGSS-----G 235
hPiezo1         -----------LQEAATLAPTRRSR-LAARFRVTAHWLLVAAGRVLAVTLLALA-----G 212
hPiezo2         DFNGGDGVEGELEESTKLKMFRRLASVASKLKEFIGNMITTAGKVVVTILLGSS-----G 235
dPiezo          ---------GDVQQATVTTPLQQQQQQLRKRVSMISQHIHFEGLVKISPLFCLATLFFAA 225

mPiezo1         IAHPSAFSSIYLVVFLAICTWWSCHFPLSPLGFNTLCVMVSCFGAGHLICLYCYQTPFIQ 279
mPiezo2         MMLPSLTSAVYFFVFLGLCTWWSWCRTFDPLLFGCLCVLLAIFTAGHLIGLYLYQFQFFQ 295
hPiezo1         IAHPSALSSVYLLLFLALCTWWACHFPISTRGFSRLCAAVGCFGAGHLICLYCYQMPLAQ 272
hPiezo2         MMLPSLTSSVYFFVFLGLCTWWSWCRTFDPLLFSCLCVLLAIFTAGHLIGLYLYQFQFFQ 295
dPiezo          VLRPSVPGGFYFLIFLLSGTYWATCQTLQRG-FALLLRCVMVVLVLHSLSIVSYQTPWMQ 284

mPiezo1         DMLPPGNIWARLFGLKNFVDLPNYSSPNALVLNTKHAWPIYVSPGILLLLYYTATSLLKL 339
mPiezo2         EAVPPNDYYARLFGIKSVIQT-DCASTWKIIVNPDLSWYHHANPILLLVMYYTLATLIRI 354
hPiezo1         ALLPPAGIWARVLGLKDFVGPTNCSSPHALVLNTGLDWPVYASPGVLLLLCYATASLRKL 332
hPiezo2         EAVPPNDYYARLFGIKSVIQT-DCSSTWKIIVNPDLSWYHHANPILLLVMYYTLATLIRI 354
dPiezo          SHLNHTTLTARLIGLEPLIESYCSPDIRVFLYNNKLSLDSYLNPFALFFAYFALALTTKH 344

mPiezo1         HKSCPSELRKETPRED-----------EEHELELDHLEPEPQAR-----------DATQG 377
mPiezo2         WLQEPLVQEEMAKEDEGALDCSSNQNTAERRRSLWYATQYPTDERKLLSMTQDDYKPSDG 414
hPiezo1         RAYRPSGQRKEAAKGY-----------EARELELAELDQWPQER-----------ESDQH 370
hPiezo2         WLQEPLVQDEGTKEEDKALACSPIQITAGRRRSLWYATHYPTDERKLLSMTQDDYKPSDG 414
dPiezo          LIKPRLVRQSTRKART---------------------PQPLES-----------GSSVA 371

mPiezo1         EMPMTTEPDLDNCTVH---------------------VLTSQSPVRQRPVRPRLAELKE 415
mPiezo2         LLVTVNGNPVDYHTIHPSLPIENGPAKTDLYTTPQYRWEPSEESSEKKEEEEDKREDSEG 474
hPiezo1         VVPTAPDTEADNCIVH---------------------ELTGQSSLLRRPVRPKRAEPGE 408
hPiezo2         LLVTVNGNPVDYHTIHPSLPMENGPGKADLYSTPQYRWEPSDESSEKREEEEEEKEEFEE 474
dPiezo          PSVTQRGNDMQLESME-------------------------QRSEQENTTTSILDQISY 405

mPiezo1         -----------MSPLHGLGHLIMDQSYVCALIAMMVWSIMYHSWLTFVLLLWACLIWTVR 464
mPiezo2         EGSQEEKRSVRMHAMVAVFQFIMKQSYICALIAMMAWSITYHSWLTFVLLIWSCTLWMIR 534
hPiezo1         -----------ASPLHSLGHLIMDQSYVCALIAMMVWSITYHSWLTFVLLLWACLIWTVR 457
hPiezo2         ERSREEKRSIKVHAMVSVFQFIMKQSYICALIAMMAWSITYHSWLTFVLLIWSCTLWMIR 534
dPiezo          G------------FVSVGGFIYQNSYIFTNILMMAWSIVYHSWLTFVLLLSANVLWMIP 452

mPiezo1         SRHQLAMLCSPCILLYGLTLCCLRYVWAMEL--PELPTTLGPVSL------HQLGLEHTR 516
mPiezo2         NRRKYAMISSPFMVVYANLLLVLQYIWSFEL--PEIKKVPGFLEK------KEPG----- 581
hPiezo1         SRHQLAMLCSPCILLYGMTLCCLRYVWAMDLR-PELPTTLGPVSL------RQLGLEHTR 510
hPiezo2         NRRKYAMISSPFMVVYGNLLLILQYIWSFEL--PEIKKVPGFLEK------KEPG----- 581
dPiezo          NQRKAMMRSSPFIVLYAEALLIAQYIYGMDLNNEELPTSVPTAGINLQQIGFERPIENQM 512

mPiezo1         YPCLDLGAMLLYLLTFWLLLRQFVKEKLLKKQKVPAALLEVTVADTEPTQT--------- 567
mPiezo2         ----ELASKILFTITFWLLLRQHLTEQKALREKE-ALLSEVKIGSQELEEKEDEELQDVQ 636
hPiezo1         YPCLDLGAMLLYTLTFWLLLRQFVKEKLLKWAESPAALTEVTVADTEPTRT--------- 561
hPiezo2         ----ELASKILFTITFWLLLRQHLTEQKALQEKE-ALLSEVKIGSQENEEK-DEELQDIQ 635
dPiezo          RPCVPLIVKTAFVLMFWVTSRQFFKEKRDRRRDSTLADFIAPLQITVGSAG--------- 563

mPiezo1         -----------------------------------QTLLRSLGELVTGIYVKYWIYVCA 591
mPiezo2         VEGEPTEKEEEEEEEIKEERHEVKKEEEEEVEEDDDQDIMKVLGNLVVALFIKYWIYVCG 696
hPiezo1         -----------------------------------QTLLQSLGELVKGVYAKYWIYVCA 585
hPiezo2         VEGEPKE---EEEEEAKEEKQERKKVEQEEAEEEEDEQDIMKVLGNLVVAMFIKYWIYVCG 692
dPiezo          ------------------------SSYLINDGKKTSKFLKKAGDVIKNLLVRLWIWLLV 598
```

```
mPiezo1          GMFIVVSFAGR-LVVYKIVYMFLFLLCLTLFQVYYTLWRKLLRVFWWLVVAYTMLVLIAV 650
mPiezo2          GMFFFVSFEGK-IVMYKIIYMVLFLFCVALYQVHYEWWRKILKYFWMSVVIYTMLVLIFI 755
hPiezo1          GMFIVVSFAGR-LVVYKIVYMFLFLLCLTLFQVYYSLWRKLLKAFWWLVVAYTMLVLIAV 644
hPiezo2          GMFFFVSFEGK-IVMYKIIYMVLFLFCVALYQVHYEWWRKILKYFWMSVVIYTMLVLIFI 751
dPiezo           LVIFLCAITGENMTGFRICYMALFLFFLLVFQSSSKAWVKIMYGFWLFLIFYAMSILILI 658

mPiezo1          YTFQFQDFPTYWRNLTGFTDEQLGDLGLEQFSVSELFSSILIPGFFLLACILQLHYFHRP 710
mPiezo2          YTYQFENFPGLWQNMTGLKKEKLEDLGLKQFTVAELFTRIFIPTSFLLVCILHLHYFHDR 815
hPiezo1          YTFQFQDFPAYWRNLTGFTDEQLGDLGLEQFSVSELFSSILVPGFFLLACILQLHYFHRP 704
hPiezo2          YTYQFENFPGLWQNMTGLKKEKLEDLGLKQFTVAELFTRIFIPTSFLLVCILHLHYFHDR 811
dPiezo           YTYQFDKFDTYWSDYLNVSATLQKDIGLKRYQTKDLFLHLVSPTIIVILTVIQVHYFHKR 718

mPiezo1          FMQLTDLEHVPPP--GTRHP-------------RWAHRQDAVSEAPLLEHQ--------- 746
mPiezo2          FLELTDLKSIPSKEDNTIYSHAKVNGRVYLIINRLAHPEGSLPDLAIMNMTASLDKPEVQ 875
hPiezo1          FMQLTDMEHVSLP--GTRLP-------------RWAHRQDAVSGTPLLREEQQEHQQQQQ 749
hPiezo2          FLELTDLKSIPSKEDNTIY--------------RLAHPEGSLPDLTMMHLTASLEKPEVR 857
dPiezo           FIASLQQQPLAGG----------------------SAQQKPTETTALEPAPSKRRGSAG 755

mPiezo1          ---EEEEVF-------------------REDGQSMDGPHQATQVPEGTASKWGLVADRLL 784
mPiezo2          KLAESGEERPEECVKKTEKGEAGKDSDESEEEEDEEEESEEE-SSDLRNKWHLVIDRLT 934
hPiezo1          EEEEEEEDS-------------------RDEGLGVATPHQATQVPEG-AAKWGLVAERLL 789
hPiezo2          KLAEPGEEKLEGYSEKAQKGDLGKDSEESEEDGEEEEESEEEEETSDLRNKWHLVIDRLT 917
dPiezo           SLRKSQGPS--------------------------AEAAPGATTDFETSVRDLVRISFR 788

mPiezo1          DLAASFSAVLTRIQVFVRRLLELHVFKLVALYTVWVALKEVSVMNLLLVVLWAFALP-YP 843
mPiezo2          VLFLKFLEYFHKLQVFMWWILELHIIKIVSSYIIWVTVKEVSLFNYVFLISWAFALP-YA 993
hPiezo1          ELAAGFSDVLSRVQVFLRRLLELHVFKLVALYTVWVALKEVSVMNLLLVVLWAFALP-YP 848
hPiezo2          VLFLKFLEYFHKLQVFMWWILELHIIKIVSSYIIWVSVKEVSLFNYVFLISWAFALP-YA 976
dPiezo           KIKNKSEYIFKNFKDVFWRFLELHIMKAVYIAAFVCSVSEVCVLHIIFVGFCVLGATSRK 848

mPiezo1          RFRPMASCLSTVWTCIIIVCKMLYQLKIVNPHEYSSNCTEPFPNNTNLQPLEINQSLLYR 903
mPiezo2          KLRRAASSVCTVWTCVIIVCKMLYQLQTIKPENFSVNCSLPNENQTNIPLHELNKSLLYS 1053
hPiezo1          RFRPMASCLSTVWTCVIIVCKMLYQLKVVNPQEYSSNCTEPFPNSTNLLPTEISQSLLYR 908
hPiezo2          KLRRLASSVCTVWTCVIIVCKMLYQLQTIKPENFSVNCSLPNENQTNIPFNELNKSLLYS 1036
dPiezo           AVQVVISRLISFIVTVIVLSKMIYQIEYLSHSQHNVVCSD-NRTANNAEWIGLTKADKVT 907

mPiezo1          GPVDPANWFGVRKGYPNLGYIQNHLQILLLLVFEAVVYRRQEHYRRQH-QQAPLPAQAVC 962
mPiezo2          APVDPTEWVGLRKSSPLLVYLRNNLLMLAILAFEVTVYRHQEYYRGRN-NLTAPVSKTIF 1112
hPiezo1          GPVDPANWFGVRKGFPNLGYIQNHLQVLLLLVFEAIVYRRQEHYRRQH-QLAPLPAQAVF 967
hPiezo2          APIDPTEWVGLRKSSPLLVYLRNNLLMLAILAFEVTIYRHQEYYRGRN-NLTAPVSRTIF 1095
dPiezo           G--------------GLMSLLRTYIIYMVIVTMHAVISLRQLQMRVKIGALNAPPTKLLF 953

mPiezo1          ADGTRQRLDQDLLSCLKYFINFFFYKFGLEICFLMAVNVIGQRMNFMVILHGCWLVAILT 1022
mPiezo2          HDITRLHLDDGLINCAKYFVNYFFYKFGLETCFLMSVNVIGQRMDFYAMIHACWLIGVLY 1172
hPiezo1          ASGTRQQLDQDLLGCLKYFINFFFYKFGLEICFLMAVNVIGQRMNFLVTLHGCWLVAILT 1027
hPiezo2          HDITRLHLDDGLINCAKYFINYFFYKFGLETCFLMSVNVIGQRMDFYAMIHACWLIAVLY 1155
dPiezo           PNIIRADAEKDLVGLVKYLLNFGFYKFGIEISLIALVSTITYRQDIVAVVYALWLVVLLL 1013

mPiezo1          RRRREAIARLWPNYCLFLTLFLLYQYLLCLGMPPALCIDYPWRWSKAIPMNSALIKWLYL 1082
mPiezo2          RRRRKAIAEVWPKYCCFLACIITFQYFVCIGIPPAPCRDYPWRFKGAY-FNDNIIKWLYF 1231
hPiezo1          RRHRQAIARLWPNYCLFLALFLLYQYLLCLGMPPALCIDYPWRWSRAVPMNSALIKWLYL 1087
hPiezo2          RRRRKAIAEIWPKYCCFLACIITFQYFICIGIPPAPCRDYPWRFKGAS-FNDNIIKWLYF 1214
dPiezo           LR-RSQCAKIWGVFQAFFAISILTQYIVLVGLPPSSCLVFPWDEGPFG---EGIQRWAML 1069

mPiezo1          PDFFRAPNSTNLISDFLLLLCASQQWQVFSAERTEEWQRMAGINTDHLEPLRGE------ 1136
mPiezo2          PDFIVRPNPVFLVYDFMLLLCASLQRQIFEDENKAAVRIMAGDNVEICMNLDAASF---S 1288
hPiezo1          PDFFRAPNSTNLISDFLLLLCASQQWQVFSAERTEEWQRMAGVNTDRLEPLRGE------ 1141
hPiezo2          PDFIVRPNPVFLVYDFMLLLCASLQRQIFEDENKAAVRIMAGDNVEICMNLDAASF---S 1271
dPiezo           PGALHFNHVPKLIFDFIVLVILNRQKSIFCIEQRYASNDDYPGGSNRSVIADIAQLGRVP 1129

mPiezo1          -PNPIPNFIHCR-SYLDMLKVAVFRYLFWLVLVVVFVAGATRISIFGLGYLLACFYLLLF 1194
mPiezo2          QHNPVPDFIHCR-SYLDMSKVIIFSYLFWFVLTIIFITGTTRISIFCMGYLVACFYFLLF 1347
hPiezo1          -PNPVPNFIHCR-SYLDMLKVAVFRYLFWLVLVVVFVTGATRISIFGLGYLLACFYLLLF 1199
hPiezo2          QHNPVPDFIHCR-SYLDMSKVIIFSYLFWFVLTIIFITGTTRISIFCMGYLVACFYFLLF 1330
dPiezo           FDNPTHDFCSYIRNYSDILKNGVLCGFYWFTLAVVFLAGTNIADLLALGYLIGAFIFLWQ 1189

mPiezo1          GTTLLQKDTRAQLVLWDCLILYNVTVIISKNMLSLLSCVFVEQMQSNFCWVIQLFSLVCT 1254
mPiezo2          GGDLLLKPIKSILRYWDWLIAYNVFVITMKNILSIGACGYIGALVRNSCWLIQAFSLACT 1407
hPiezo1          GTALLQRDTRARLVLWDCLILYNVTVIISKNMLSLLACVFVEQMQTGFCWVIQLFSLVCT 1259
hPiezo2          GGDLLLKPIKSILRYWDWLIAYNVFVITMKNILSIGACGYIGTLVHNSCWLIQAFSLACT 1390
dPiezo           GSDFYLRPIHTIIFRWKWLLAFNVANILIKTSFQMAGCLFMTQLTKDCCWLVHMLGITCT 1249
```

```
mPiezo1         VKGYYDPKEMMTRDRDCLLP------VEEAGIIWDSICFFFLLLQRRIFLSHYFLHVSAD 1308
mPiezo2         VKGYQMP----EDDSRCKLP------SGEAGIIWDSICFAFLLLQRRVFMSYYFLHVVAD 1457
hPiezo1         VKGYYDPKEMMDRDQDCLLP------VEEAGIIWDSVCFFFLLLQRRVFLSHYYLHVRAD 1313
hPiezo2         VKGYQMP----AANSPCTLP------SGEAGIIWDSICFAFLLLQRRVFMSYYFLHVVAD 1440
dPiezo          SNVLTEQIMLPEEAELALKPGECPKITHQVVLLWDTICFAFIIFQLRIFKSHYFCHIITD 1309

mPiezo1         LKATALQASRGFALYNAANLKSINFHRQIEEKSLAQLKRQMKRIRAKQEKYRQSQ----- 1363
mPiezo2         IKASQILASRGAELFQATIVKAVKARIEEEKKSMDQLKRQMDRIKARQQKYKKGKERMLS 1517
hPiezo1         LQATALLASRGFALYNAANLKSIDFHRRIEEKSLAQLKRQMERIRAKQEKHRQGR----- 1368
hPiezo2         IKASQILASRGAELFQATIVKAVKARIEEEKKSMDQLKRQMDRIKARQQKYKKGKERMLS 1500
dPiezo          TKANNILASRGADIIESLRHKQIAHRHDHEKQVLHKIKRKMERIRATQQKMLRPLDKQTH 1369

mPiezo1         --ASRGQLQ-SKDPQDPSQEPGPDSPGGSSPPRRQWWRPWLDHATVIHSGDYFLFESDSE 1420
mPiezo2         LTQESGEGQDIQKVSEEDDEREADKQKAKGK-KKQWWRPWVDHASMVRSGDYYLFETDSE 1576
hPiezo1         --VDRSRPQDTLGPKDPGLEPGPDSPGGSSPPRRQWWRPWLDHATVIHSGDYFLFESDSE 1426
hPiezo2         LTQEPGEGQDMQKLSEEDDEREADKQKAKGK-KKQWWRPWVDHASMVRSGDYYLFETDSE 1559
dPiezo          FDEHGYPLPAPTVRRRKEIKLHPHATRAGDYYMFEEMDDKFELDLIHDEIDFLEEENITE 1429

mPiezo1         EEEE-------ALPEDPRPAAQSAFQMAYQAWVTNAQTVLRQRR---ERARQERAEQLAS 1470
mPiezo2         EEEEEE-----LKKEDEEPPRKSAFQFVYQAWITDPKTALRQRRKEKKKLAREEQKERRK 1631
hPiezo1         EEEE-------AVPEDPRPSAQSAFQLAYQAWVTNAQAVLRRRQQEQEQARQEQAGQLPT 1479
hPiezo2         EEEEEE-----LKKEDEEPPRRSAFQFVYQAWITDPKTALRQRHKEKKRSAREERKRRRK 1614
dPiezo          SEMKMQRRKTLYDKSKDAPTGEFPSTSKGISKERDAATASSSASPAPTRDVGDLPVIPPP 1489

mPiezo1         G---GDLNPDVEPVDVPEDEMAGRSHMMQRVLS---TMQFLWVLGQATVDGLTRWLRAFT 1524
mPiezo2         GSGDGPVEWEDREDEPVKKKSDGPDNIIKRIFN---ILKFTWVLFLATVDSFTTWLNSIS 1688
hPiezo1         G---GGPSQEVEPAEGPEEAAAGRSHVVQRVLS---TAQFLWMLGQALVDELTRWLQEFT 1533
hPiezo2         GSKEGPVEWEDREDEPIKKKSDGPDNIIKRIFN---ILKFTWVLFLATVDSFTTWLNSIS 1671
dPiezo          STGLGREQTSKETSDSKSKMEVDSGEVTAKDSDEDFDTNPIIRLLEGFLVTLTIRLNRFS 1549

mPiezo1         KHHRTMSDVLCAERYLLTQ--ELLRVGEVRRGVLDQLYVGEDE-ATLSGPVETRDGPSTA 1581
mPiezo2         REHIDISTVLRIERCMLTR--EIKKGNVPTRESIHMYYQNHIMNLSRESGLDTIDEHSGA 1746
hPiezo1         RHHGTMSDVLRAERYLLTQ--ELLQGGEVHRGVLDQLYTSQAE-ATLPGPTEAPNAPSTV 1590
hPiezo2         REHIDISTVLRIERCMLTR--EIKKGNVPTRESIHMYYQNHIMNLSRESGLDTIDEHPGA 1729
dPiezo          RNYRFVNRILAGEKKTLKESSSLNRLGLSSAAAMFHFLKSNLESDESEPPASSSTPRRVV 1609

mPiezo1         SSGLGAEEPLSSMTDDTSSP---------------------------------------- 1601
mPiezo2         GSRAQAAHRMDSLDSRDSISSCYTEATLLISRQSTLDDLDGQDPVPKTSERARPRLRKMF 1806
hPiezo1         SSGLGAEEPLSSMTDDMGSP---------------------------------------- 1610
hPiezo2         ASGAQTAHRMDSLDSHDSIS---------------------------------------- 1749
dPiezo          IAPPNATEHSDPTSTTLNTN---------------------------------------- 1629

mPiezo1         --------------------LSTGYNTRSGSEEIVTDAGDLQ------------------ 1623
mPiezo2         SLDMSSSSADSGSVASSEPTQCTMLYSRQGTTETIEEVEAEAEEEVVEGLEPELHDAEEK 1866
hPiezo1         --------------------LSTGYHTRSGSEEAVTDPGERE------------------ 1632
hPiezo2         ----------------SEPTQCTMLYSRQGTTETIEEVEAEQEEEAGS-TAPEPREAKEY 1792
dPiezo          -------------------TTTTPLSPPEPLQPLQPNTTSTPQQ---------------- 1654

mPiezo1         ------------AGTSLHGSQEL------------------------------------- 1634
mPiezo2         EYA--AEYEAGVEEISLTPDEELPQFSTDD--CEAPPSYSKAVSFEHLSFASQDDSGAKN 1922
hPiezo1         ------------AGASLYQG---------------------------------------- 1640
hPiezo2         EATGYDVGAMGAEEASLTPEEELTQFSTLDGDVEAPPSYSKAVSFEHLSFGSQDDSAGKN 1852
dPiezo          ------------QHQHIRAAEEIIELPVDT------------------------------ 1672

mPiezo1         -----------------LANARTRMRTASELLLDRRLHIPELEEAERFEAQQGRTLRLLR 1677
mPiezo2         HMVVSPDDSRTDKLESSILPPLTHELTASDLLMSKMFHDDELEESEKFYVDQPRFLLLFY 1982
hPiezo1         -----------------------LMRTASELLLDRRLRIPELEEAELFAEGQGRALRLLR 1677
hPiezo2         RMAVSPDDSRTDKLGSSILPPLTHELTASELLLKKMFHDDELEESEKFYVGQPRFLLLFY 1912
dPiezo          -----------------VDGVAHRKQSINSSPPAKGAGEFNLEEENFAQRDHHIIVEVLI 1715

mPiezo1         AGYQCVAAHSELLCYFIIILNHMVTASAASLVLPVLVFLWAMLTIPRPSKRFWMTAIVFT 1737
mPiezo2         AMYNTLVARSEMVCYFVIILNHMTSASIITLLLPILIFLWAMLSVPRPSRRFWMMAIVYT 2042
hPiezo1         AVYQCVAAHSELLCYFIIILNHMVTASAGSLVLPVLVFLWAMLSIPRPSKRFWMTAIVFT 1737
hPiezo2         AMYNTLVARSEMVCYFVIILNHMVSASMITLLLPILIFLWAMLSVPRPSRRFWMMAIVYT 1972
dPiezo          SSWYALLANTDLICYIVVFINQVVNASLISLPLPIMVFLWGTLSLPRPTKTFWVTLIAYT 1775

mPiezo1         EVMVVTKYLFQFGFFPWNSYVVLRRYENKPYFPPRILGLEKTDSYIKYDLVQLMALFFHR 1797
mPiezo2         EVAIVVKYFFQFGFFPWNKDLEIY--KERPYFPPNIIGVEKKEGYVLYDLIQLLALFFHR 2100
hPiezo1         EIAVVVKYLFQFGFFPWNSHVVLRRYENKPYFPPRILGLEKTDGYIKYDLVQLMALFFHR 1797
hPiezo2         EVAIVVKYFFQFGFFPWNKNVEVN--KDKPYHPPNIIGVEKKEGYVLYDLIQLLALFFHR 2030
dPiezo          QAIVLIKCIFQFKLIWSNYHQLPN----QPLTPAKIFGVENKAHYAIYDLILLLVLFLHR 1831
```

```
mPiezo1         SQLLCYGLWDHEEDRYPKDHCRSSVKDREAKEEPEAKLESQSETGTGHPKEPVLAGTPRD 1857
mPiezo2         SILKCHGLWDEDDIVDSNTDKEGSDDELSLDQGR-----------------RGSSDSLK 2142
hPiezo1         SQLLCYGLWDHEEDSPSKEHDKSGEEEQGAEEGP----------------GVPAATTED 1840
hPiezo2         SILKCHGLWDEDDMTESGMAREESDDELSLGHGR-----------------RDSSDSLK 2072
dPiezo          YLLKSQGLWKSGYKDTDNQFTKPTASIDERDDSD------------------------- 1865

mPiezo1         HIQGKGSIRSKDVIQDPPEDLKPRHTRHISIRFRRRK-ETPGPKGTAVMETEHEEGEGKE 1916
mPiezo2         SINLAASVESVHVTFPEQPAAIRRKRSCSSSQISPRSSFSSNRSKRGSTSTRNSSQKGSS 2202
hPiezo1         HIQVEARVGPTDGTPEPQVELRPRDTRRISLRFRRRKKEGPARKGAAAIEAEDREEEEGE 1900
hPiezo2         SINLAASVESVHVTFPEQQTAVRRKRSGSSSEPSQRSSFSSNRSQRGSTSTRNSSQKGSS 2132
dPiezo          -----------NLSQPDSRQLNDDAAQKLSLQVSQASLPGSPEFSKTGINQLERTKYTSS 1914

mPiezo1         TTERKRPRHTQEKSKFRERMKAAGRRLQSFCVSLAQSFYQPLQRFFHDILHTKYRAATDV 1976
mPiezo2         VLSLK---QKSKRELYMEKLQEHLIKAKAFTIKKTLQIYVPIRQFFYDLIHPDYSAVTDV 2259
hPiezo1         EEKEAPTGREKRPSRSGGRVRAAGRRLQGFCLSLAQGTYRPLRRFFHDILHTKYRAATDV 1960
hPiezo2         VLSIK---QKGKRELYMEKLQEHLIKAKAFTIKKTLEIYVPIKQFFYNLIHPEYSAVTDV 2189
dPiezo          ---------------------------------------LYKFFFSLVHKSR-LATDV 1932

mPiezo1         YALMFLADIVDIIIIIFGFWAFGKHSAATD--IASSLSDDQVPQAFLFMLLVQFGTMVID 2034
mPiezo2         YVLMFLADTVDFIIIVFGFWAFGKHSAAAD--ITSSLSEDQVPGPFLVMVLIQFGTMVVD 2317
hPiezo1         YALMFLADVVDFIIIIFGFWAFGKHSAATD--ITSSLSDDQVPEAFLVMLLIQFSTMVVD 2018
hPiezo2         YVLMFLADTVDFIIIVFGFWAFGKHSAAAD--ITSSLSEDQVPGPFLVMVLIQFGTMVVD 2247
dPiezo          YALMFLCDFVNFFVLLFGFTAFGTQQTESDEGVQTYLAENKVPIPFLIMLLVQFLLIVID 1992

mPiezo1         RALYLRKTVLGKLAFQVVLVVAIHIWMFFILPAVTERMFSQNAVAQLWYFVKCIYFALSA 2094
mPiezo2         RALYLRKTVLGKVIFQVILVFGIHFWMFFILPGVTERKFSQNLVAQLWYFVKCVYFGLSA 2377
hPiezo1         RALYLRKTVLGKLAFQVALVLAIHLWMFFILPAVTERMFNQNVVAQLWYFVKCIYFALSA 2078
hPiezo2         RALYLRKTVLGKVIFQVILVFGIHFWMFFILPGVTERKFSQNLVAQLWYFVKCVYFGLSA 2307
dPiezo          RALYLRKALVNKIIFHFFSVIGIHIWMFFVVPAVTERTFNSLAPPIIFYVIKCFYMLLSS 2052

mPiezo1         YQIRCGYPTRILGNFLTKKYNHLNLFLFQGFRLVPFLVELRAVMDWVWTDTTLSLSNWMC 2154
mPiezo2         YQIRCGYPTRVLGNFLTKSYNYVNLFLFQGFRLVPFLTELRAVMDWVWTDTTLSLSSWIC 2437
hPiezo1         YQIRCGYPTRILGNFLTKKYNHLNLFLFQGFRLVPFLVELRAVMDWVWTDTTLSLSSWMC 2138
hPiezo2         YQIRCGYPTRVLGNFLTKSYNYVNLFLFQGFRLVPFLTELRAVMDWVWTDTTLSLSSWIC 2367
dPiezo          YQIKSGYPKRILGNFFTKGFSMVNMIAFKVYMQIPFLYELRTILDWVCIDSTMTIFDWLK 2112

mPiezo1         VEDIYANIFIIKCSRETEKKYPQPKGQKKKKIVKYGMGGLIILFLIAIIWFPLLFMSLIR 2214
mPiezo2         VEDIYAHIFILKCWRESEKRYPQPRGQKKKKAVKYGMGGMIIVLLICIVWFPLLFMSLIK 2497
hPiezo1         VEDIYANIFIIKCSRETEKKYPQPKGQKKKKIVKYGMGGLIILFLIAIIWFPLLFMSLVR 2198
hPiezo2         VEDIYAHIFILKCWRESEKRYPQPRGQKKKKVVKYGMGGMIIVLLICIVWFPLLFMSLIK 2427
dPiezo          MEDIFSNIYLIRCTRQSETDFPAMRAQKKASLSKLIMGGTIVLLIVICIWGPLCLFALGN 2172

mPiezo1         SVVGVVNQPIDVTVTLKLGGYEPLFTMSAQQPSIVPFTPQAYEELSQQFDPYPLAMQFIS 2274
mPiezo2         SVAGVINQPLDVSVTITLGGYQPIFTMSAQQSQLKVMDNSKYNEFLKSFGPNSGAMQFLE 2557
hPiezo1         SVVGVVNQPIDVTVTLKLGGYEPLFTMSAQQPSIIPFTAQAYEELSRQFDPQPLAMQFIS 2258
hPiezo2         SVAGVINQPLDVSVTITLGGYQPIFTMSAQQSQLKIMDQQSFNKFIQAFSRDTGAMQFLE 2487
dPiezo          -AVGTSNVPFHVSLSIRIGPYDPIYTTNN-YDSIFEINPEMYSQMTNAYIKEKQALTFIA 2230

mPiezo1         QYSPEDIVTAQIEGSSGALWRISPPSRAQMKQELYNGTADITLRFTWNFQRDLAKGGTVE 2334
mPiezo2         NYEREDVTVAELEGNSNSLWTISPPSKQKMIQELTDPNSCFSVVFSWSIQRNMTLGAKAE 2617
hPiezo1         QYSPEDIVTAQIEGSSGALWRISPPSRAQMKRELYNGTADITLRFTWNFQRDLAKGGTVE 2318
hPiezo2         NYEKEDITVAELEGNSNSLWTISPPSKQKMIHELLDPNSSFSVVFSWSIQRNLSLGAKSE 2547
dPiezo          GYDATDVAAVRLAGNSPSLWNIAPPDRQRLLNDLRN-NHTLKARFSYSLTRKAPAKGLKE 2289

mPiezo1         YTNEKHTLELAPNSTARRQLAQLLEGRPDQS------------------VVIPHLFPKYI 2376
mPiezo2         IATDKLSFPLAV--ATRNSIAKMIAGNDTESSNTP--------------VTIEKIYPYYV 2661
hPiezo1         YANEKHMLALAPNSTARRQLASLLEGTSDQS------------------VVIPNLFPKYI 2360
hPiezo2         IATDKLSFPLKN--ITRKNIAKMIAGNSTESSKTP--------------VTIEKIYPYYV 2591
dPiezo          NVGDEHAISLDESFEGRAALIHMLSETHDVEPIHSNGTTNGTTPEVEEVVVIPGMIPKFI 2349

mPiezo1         RAPNGPEANPVKQLQPDEEEDYLGVRIQLRREQVGTGASGEQAGTKASDFLEWWVIELQD 2436
mPiezo2         KAPSDSNSKPIKQLLS--ENNFMNITIIILFRDNVTKSNSE------------WWVLNLTG 2707
hPiezo1         RAPNGPEANPVKQLQPNEEADYLGVRIQLRREQ-GAGATG---------FLEWWVIELQE 2410
hPiezo2         KAPSDSNSKPIKQLLS--ENNFMDITIILSRDNTTKYNSE------------WWVLNLTG 2637
dPiezo          KVLNSGDAAVVSVLSP-KHYDYRPLVIKMHRDNETNGLWWEIRDYCN----DTFYNETLS 2404

mPiezo1         CKADCN---LLPMVIFSDKVSPPSLGFLAGYGIVGLYVSIVLVVGKFVRGFFSEISHSIM 2493
mPiezo2         SRIFNQGSQALELVVFNDKVSPPSLGFLAGYGIMGLYASVVLVIGKFVREFFSGISHSIM 2767
hPiezo1         CRTDCN---LLPMVIFSDKVSPPSLGFLAGYGIMGLYVSIVLVIGKFVRGFFSEISHSIM 2467
hPiezo2         NRIYNPNSQALELVVFNDKVSPPSLGFLAGYGIMGLYASVVLVIGKFVREFFSGISHSIM 2697
dPiezo          KFAYSNCTSGIVMYTFNDKKFPSTFSFLTAGGIIGLYTTFVLLASRFMKSFIGGQNRKIM 2464
```

```
mPiezo1         FEELPCVDRILKLCQDIFLVRETRELELEEELYAKLIFLYRSPETMIKWTRERE------ 2547
mPiezo2         FEELPNVDRILKLCTDIFLVRETGELELEEDLYAKLIFLYRSPETMIKWTREKTN----- 2822
hPiezo1         FEELPCVDRILKLCQDIFLVRETRELELEEELYAKLIFLYRSPETMIKWTREKE------ 2521
hPiezo2         FEELPNVDRILKLCTDIFLVRETGELELEEDLYAKLIFLYRSPETMIKWTREKTN----- 2752
dPiezo          FEDLPYVDRVLQLCLDIYLVREALEFALEEDLFAKLLFLYRSPETLIKWTRPKEEYVDDD 2524

mPiezo1         ------------------------
mPiezo2         ------------------------
hPiezo1         ------------------------
hPiezo2         ------------------------
dPiezo          GDTDSIPSRMSVRRPEQLQPQQPQ 2548
```

Supplementary Figure 3. Alignment between mouse, human and fly Piezos highlights hydrophobic regions and residues of interest. Multiple sequence alignment between mPiezo1, mPiezo2, hPiezo1, hPiezo2 and dPiezo generated using ClustalW2 (http://www.ebi.ac.uk/Tools/msa/clustalw2/). Residues highlighted in grey indicate proposed hydrophobic regions in mPiezo1. Residues highlighted in cyan indicate position of myc-tags in extracellular loops. Residues highlighted in pink indicate phosphorylation sites. Residues highlighted in green indicate junction points for the following mPiezo1/dPiezo chimeras; ASRG: $dP^{1\text{-}1316}/mP1^{1316\text{-}2547}$ and $mP1^{1\text{-}1315}/dP^{1317\text{-}2548}$, FLW: $dP^{1\text{-}1752}/mP1^{1715\text{-}2547}$ and $mP1^{1\text{-}1714}/dP^{1753\text{-}2548}$ and TDV: $dP^{1\text{-}1929}/mP1^{1974\text{-}2547}$ and $mP1^{1\text{-}1973}/dP^{1930\text{-}2548}$. Residues highlighted in yellow indicate acidic residues between regions of amino acid numbers 1974-2547 that were mutated to alanine or asparagine.

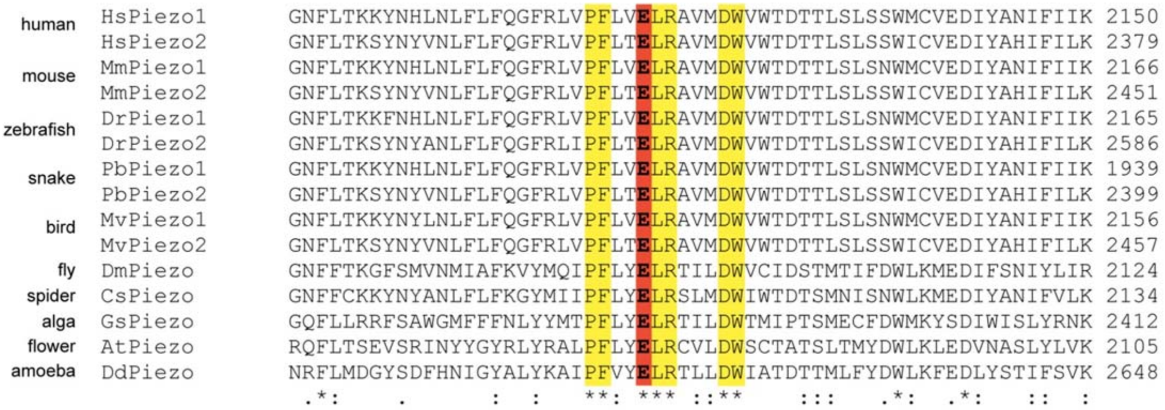

Supplementary Figure 4. Conservation of a glutamic acid residue involved in ion conduction properties of Piezo channels. Multiple sequence alignment of Piezo proteins from different species using Clustal Omega (http://www.ebi.ac.uk/Tools/msa/clustalo/). Alignment of only the region around mouse Piezo1 E2133 (red box) is shown. The yellow boxes highlight conserved residues among species close to mouse Piezo1 E2133. The sequences used for the alignment are:

Homo sapiens: HsPiezo1 (NP_001136336.2) and HsPiezo2 (NP_071351.2)

Mus musculus: MmPiezo1 (ADN28064.1) and MmPiezo2 (NP_001034574.4)

Danio rerio: DrPiezo1 (XP_696355.4) and DrPiezo2 (XP_003198010.2)

Python bivittatus: PbPiezo1 (XP_007431683.1) and PbPiezo2 (XP_007433600.1)

Manacus vitellinus: MvPiezo1 (XP_008925897.1) and MvPiezo2 (XP_008923830.1)

Drosophila melanogaster: DmPiezo (AFB77909.1)

Cupiennius salei: CsPiezo (JAA92956.1)

Galdieria sulphuraria: GsPiezo (XP_005703632.1)

Arabidopsis thaliana: AtPiezo (NP_182327.6)

Dictyostelium discoideum: DdPiezo (Q54S52.1)

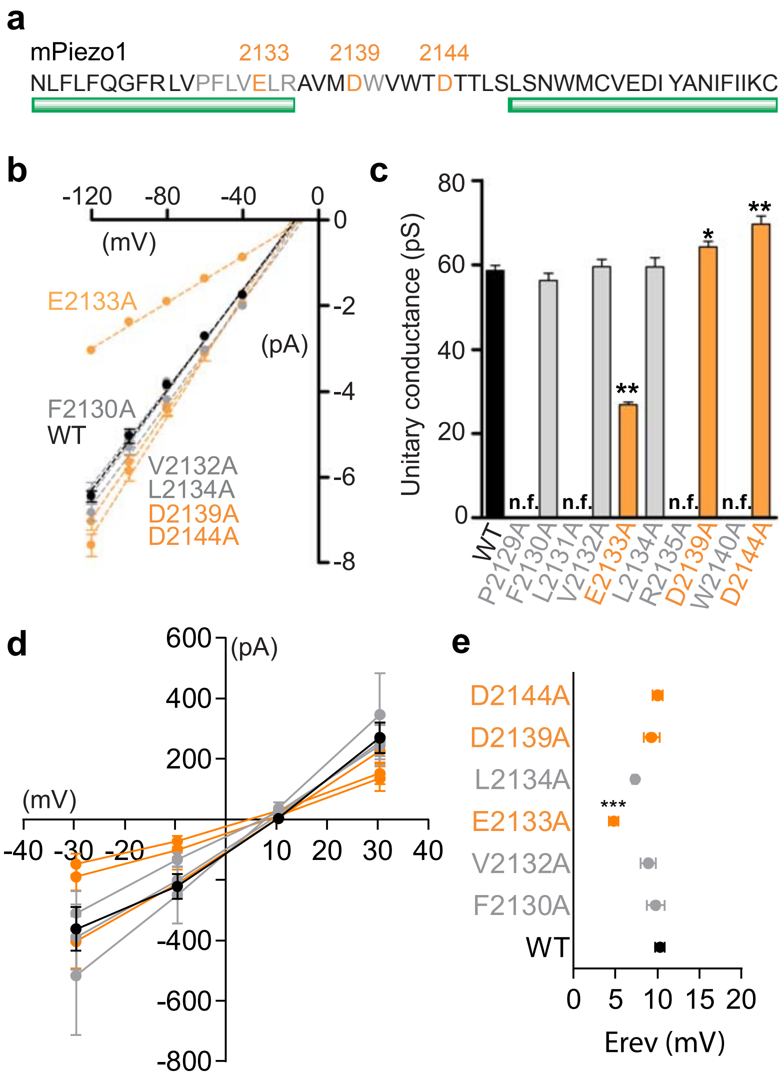

Supplementary Figure 5. Unitary conductance and ion selectivity of alanine single point mutants around E2133. (**a**) mPiezo1 sequence around E2133. Green bars indicate hydrophobic regions. E2133 and other acidic residues are highlighted in orange. Other conserved residues in this region are marked in grey. (**b**) Average I-V relationships of stretch-activated single channels in mPiezo1 WT and F2130A, V2132A, E2133A, L2134A, D2139A and D2144A transfected cells (n =7, 6, 4, 4, 5, 7, and 4, respectively; mean ± s.e.m.). Single-channel amplitude was determined as the amplitude difference in Gaussian fits of full-trace histograms. (**c**) Unitary conductance of stretch-activated channels from mPiezo1 WT and specified single point mutants transfected cells. P2129A, L2131A, R2135A and W2140A mutants are non-

functional. Conductance is calculated from the slope of linear regression line of individual cell single-channel I-V relationships (mean ± s.e.m.; One-way ANOVA with Dunn's comparison relative to WT, **P<0.01, *P<0.05). Panel b and c experiments were done in Na^+-based pipette solution. (**d**) Average I-V relationships of MA currents recorded from mPiezo1 WT, F2130A, V2132A, E2133A, L2134A, D2139A and D2144A expressing cells with 150 mM CsCl based intracellular solution and 100 mM $CaCl_2$ extracellular solution (n = 9, 5, 6, 9, 5, 7 and 7, respectively). (**e**) E_{rev} potential for the indicated mutants corresponding to panel (**d**) experiments WT: 10.3 ± 0.6 mV; F2130A: 9.8 ± 1.0 mV; V2132A: 8.9 ± 0.9 mV; E2133A: 4.8 ± 0.6 mV; L2134A: 7.3 ± 0.3 mV; D2139A: 9.3 ± 0.9 mV; D2144A: 10.0 ± 0.6 mV; mean ± s.e.m.; One-way ANOVA with Dunn's comparison to WT ***P<0.001).

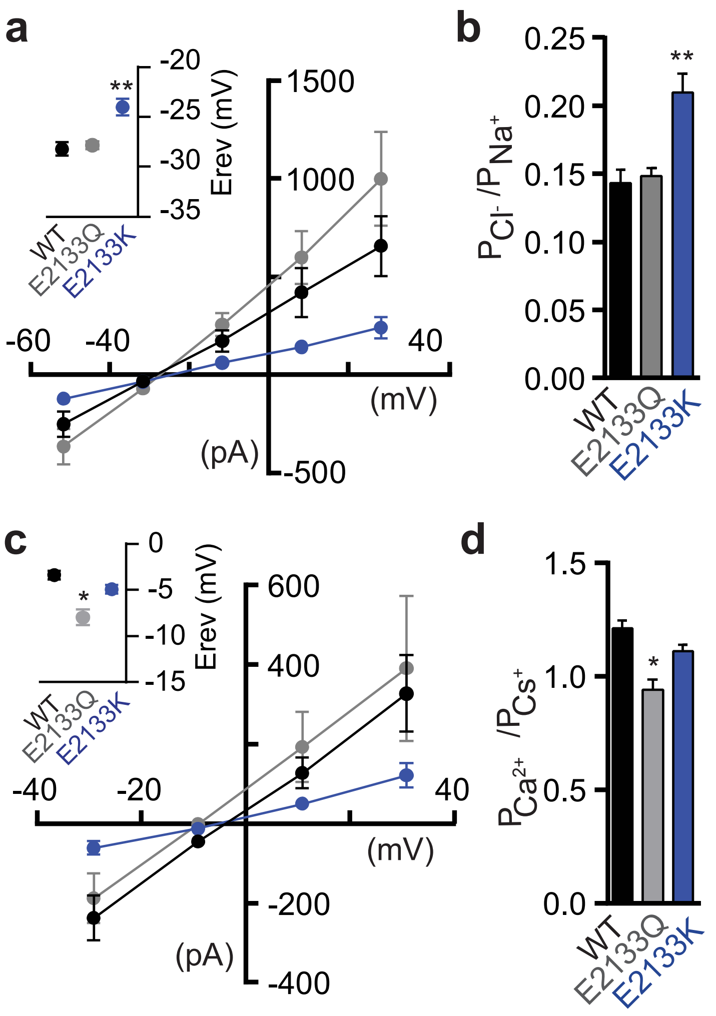

Supplementary Figure 6. Chloride and Calcium permeability for E2133 mutants. (**a**) Average I-V relationships of MA currents recorded from mPiezo1 WT, E2133Q and E2133K expressing cells with 150 mM NaCl based intracellular solution and 30 mM NaCl extracellular solution. Inset: Average reversal potential (mean ± s.e.m.; n = 6, 7 and 6, respectively; One-way ANOVA with Dunn's comparison to WT **P<0.01). (**b**) P_{Cl}/P_{Na} permeability ratios of MA currents from cells transfected with mPiezo1 WT, E2133Q and E2133K (mean ± s.e.m.; n = 6, 7 and 6, respectively; One-way ANOVA with Dunn's comparison to WT **P<0.01). (**c**) Average I-V relationships of MA currents recorded from mPiezo1 WT, E2133Q, and E2133K expressing cells with 149 mM Cs-methanesulfonate, 1 mM CsCl based intracellular solution and 50 mM Ca-Gluconate, 0.5 $CaCl_2$ based extracellular solution. Inset: Average reversal potential (mean ±

s.e.m.; n = 6, 6, and 5, respectively; One-way ANOVA with Dunn’s comparison to WT *P<0.05).

(**d**) P_{Ca}/P_{Cs} permeability ratios of MA currents from cells transfected with mPiezo1 WT, E2133Q, and E2133K (mean ± s.e.m.; n = 6, 6, and 5, respectively; One-way ANOVA with Dunn’s comparison to WT *P<0.05).

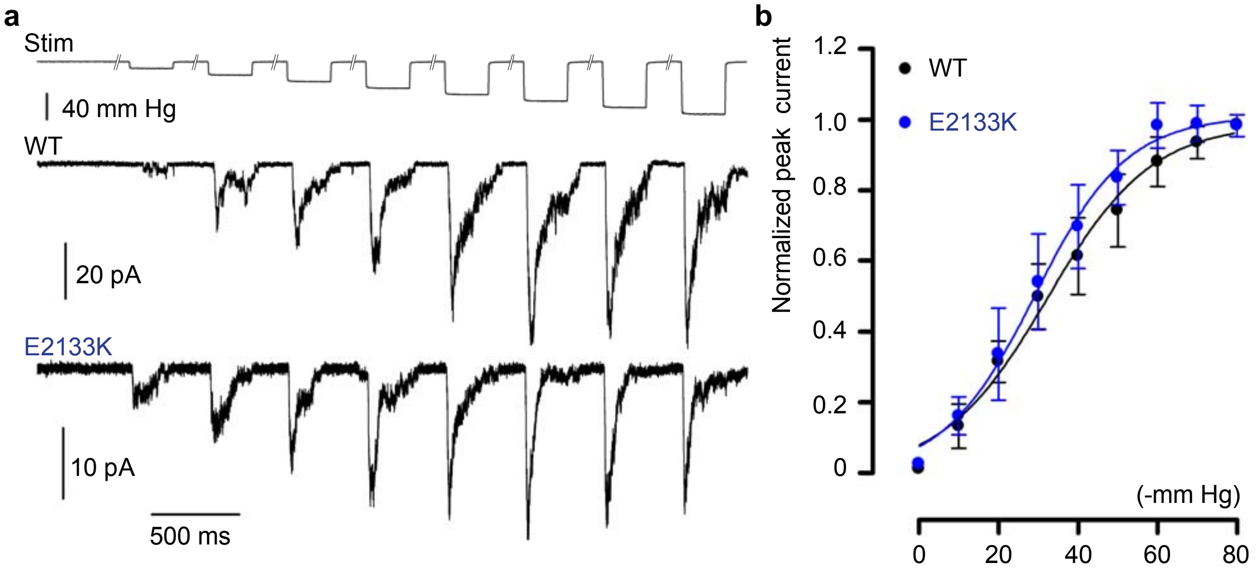

Supplementary Figure 7. Pressure sensitivity for WT and E2133K mutant. (**a**) Representative cell-attached stretch activated current traces recorded from HEK293T cells expressing WT or E2133K mPiezo1 at -80 mV with standard pipette solution (see methods). Patches are stimulated with 250-ms negative pressure pulses from 0 to -80 mmHg elicited every 10 seconds (-10 mm Hg increments). (**b**) Average peak current-pressure relationships of stretch activated currents recorded from WT or E2133K mPiezo1 expressing cells as illustrated in (**a**) (n =4 and 5 cells, respectively). Bars indicate mean ± s.e.m. Fits with boltzman equation give pressure for half maximal activation (P_{50}) of 32.3 ± 3.0 and 29.3 ± 2.9 mm Hg for WT and E2133K, respectively.

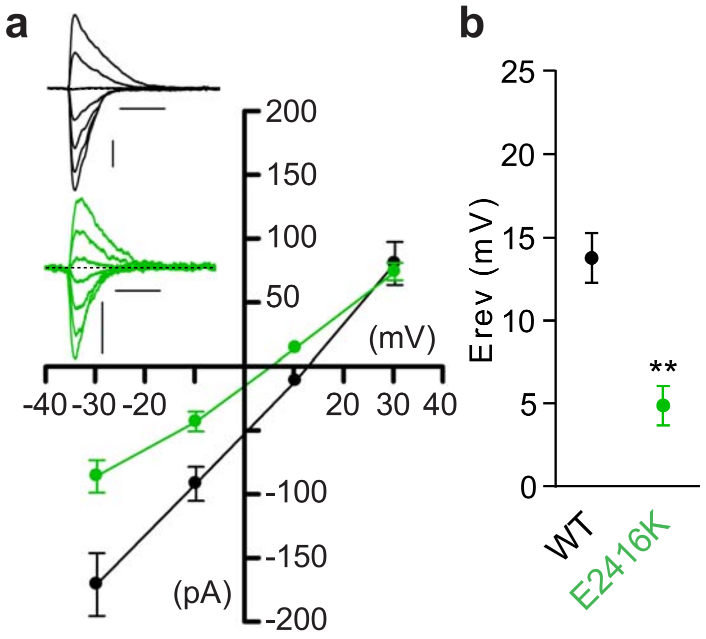

Supplementary Figure 8. Ion selectivity altered by mPiezo2 mutant. (**a**) Average I-V relationships of MA currents recorded from mPiezo2 WT and E2416K expressing cells with 150 mM CsCl based intracellular solution and 100 mM $CaCl_2$ extracellular solution (n = 6 for each). Inset: typical recording traces for WT (black) and E2416K (green) from -69.6 to +50.4 mV, Δ20 mV. Scale bars: 100 pA, 50 ms. Probe stimulation displacements are 5 and 9 µm, respectively. (**b**) Average reversal potentials from individual cells corresponding to panel (**a**) experiments (mean ± s.e.m.; Mann-Whitney test, **P<0.01).

Supplementary Table 1. Summary of Myc tag analysis used for topology prediction.

Amino acid position for Myc tag	Live labelling*
N-terminal	-
C-terminal	-
102	+
153	-
221	-
304	+
382	-
443	-
508	+
562	-
606	-
669	+
753	-
802	-
897	+
940	-
1000	-
1026	-
1071	+
1126	-
1163	-
1178	-
1204	-
1239	-
1269	-
1299	-
1389	-
1699	-
1765	+
1797	-
1891	-
1892	-
2009	-
2035	-
2041	-
2071	+
2075	+
2086	-
2104	-
2126	-
2143	-
2146	-
2157	-
2173	-
2336	+

*Negative for live labeling does not mean that the tag is intracellular.

Supplementary Table 2. Potential phosphorylated residues in peptide sequences detected by mass spectroscopy

Phosphorylated residue position	Phosphorylation residue	Peptide sequence	Total
351	T	KETPREDEEHELELDHLEPEPQAR	5
396	T	DATQGEMPMTTEPDLDNCTVHVLTSQSPVR	1
397	S	DATQGEMPMTTEPDLDNCTVHVLTSQSPVR	1
399	S	DATQGEMPMTTEPDLDNCTVHVLTSQSPVR	11
738	S	QDAVSEAPLLEHQEEEVFREDGQSMDGPHQATQVPEGTASK	1
758	S	QDAVSEAPLLEHQEEEVFREDGQSMDGPHQATQVPEGTASK	1
1385	S	DPQDPSQEPGPDSPGGSSPPR	28
		DPQDPSQEPGPDSPGGSSPPRR	23
		GQLQSKDPQDPSQEPGPDSPGGSSPPR	3
		LQSKDPQDPSQEPGPDSPGGSSPPRRQW	2
		QSKDPQDPSQEPGPDSPGGSSPPRRQW	2
		SKDPQDPSQEPGPDSPGGSSPPRRQW	1
1389	S	DPQDPSQEPGPDSPGGSSPPR	8
		DPQDPSQEPGPDSPGGSSPPRR	7
1390	S	DPQDPSQEPGPDSPGGSSPPR	5
		DPQDPSQEPGPDSPGGSSPPRR	13
		QSKDPQDPSQEPGPDSPGGSSPPRRQW	2
1593	S	DGPSTASSGLGAEEPLSSMTDDTSSPLSTGYNTR	1
1600	S	DGPSTASSGLGAEEPLSSMTDDTSSPLSTGYNTR	4
1604	T	DGPSTASSGLGAEEPLSsMTDDTSSPLSTGYNTR	2
1608	T	NTRSGSEEIVTDAGDLQAGTSLHGSQEL	1
		NTRSGSEEIVTDAGDLQAGTSLHGSQELL	1
1610	S	NTRSGSEEIVTDAGDLQAGTSLHGSQELL	2
		SGSEEIVTDAGDLQAGTSLHGSQELLANAR	2
1612	S	NTRSGSEEIVTDAGDLQAGT	2
		NTRSGSEEIVTDAGDLQAGTSL	4
		NTRSGSEEIVTDAGDLQAGTSLHGSQEL	2
		NTRSGSEEIVTDAGDLQAGTSLHGSQELL	1
		SGSEEIVTDAGDLQAGTSLHGSQELLANAR	9
1617	T	NTRSGSEEIVTDAGDLQAGTSLHGSQELL	1
1626	T	SGSEEIVTDAGDLQAGTSLHGSQELLANAR	1
1627	S	SGSEEIVTDAGDLQAGTSLHGSQELLANAR	6
1631	S	NTRSGSEEIVTDAGDLQAGTSLHGSQELL	4
		NTRSGSEEIVTDAGDLQAGTSLHGSQELLAN	1
		NTRSGSEEIVTDAGDLQAGTSLHGSQELLANARTRM	1
		SGSEEIVTDAGDLQAGTSLHGSQELLANAR	19
1640	T	NTRSGSEEIVTDAGDLQAGTSLHGSQELLANARTRM	1
1644	T	MRTASELLLDR	1
		RTASELLLDR	1
		RTASELLLDRRLHIPELEEAERF	1
1646	S	MRTASELLLDR	5
		RTASELLLDR	4
		RTASELLLDRRLHIPELEEAERF	7
		TASELLLDR	6
		TASELLLDRR	9
1837	S	LESQSETGTGHPK	7

Supplementary Table 3. Pharmacological and biophysical properties of mPiezo1/dPiezo chimeras and mPiezo mutants

	30 µM ruthenium red inhibition at -80 mV (%)	Inactivation time constant at -80mV (tau in ms)	Unitary conductance (pS)	
			Standard pipette solution	Na^+-based pipette solution
**WT channels**				
mPiezo1	71.4 ± 4.4 (5)	9.9 ± 0.5 (31)	29.1 ± 0.4 (7)	58.6 ± 1.2 (7)
mPiezo2	70.8 ± 1.4 (5)	4.5 ± 0.7 (6)	23.7 ± 0.6 (8)	56.9 ± 1.3 (9)
dPiezo	5.2 ± 6.7 (5)	6.1 ± 0.5 (6)	5.7 ± 0.6 (6)	n.d.
**Chimeras**				
$mP1^{1-1315}/dP^{1317-2548}$	Non-functional			
$dP^{1-1316}/mP1^{1316-2547}$	64.8 ± 6.7 (5)	15.9 ± 0.8 (30)	23.4 ± 1.0 (5)	n.d.
$mP1^{1-1714}/dP^{1753-2548}$	Non-functional			
$dP^{1-1752}/mP1^{1715-2547}$	76.4 ± 5.3 (6)	25.5 ± 1.3 (5)	27 ± 1 (5)	n.d.
$mP1^{1-1973}/dP^{1930-2548}$	1.3 ± 13 (5)	4.5 ± 0.5 (5)	9.1 ± 0.8 (6)	n.d.
**mPiezo1 mutants**				
D1975N	74.3 ± 4.8 (4)	7.7 ± 1.2 (9)	31.4 ± 1.1 (4)	n.d.
D1984A	78.0 ± 5.9 (4)	9.7 ± 1.2 (10)	30.2 ± 0.6 (3)	n.d.
D1987A	79.5 ± 2.5 (2)	19.1 ± 1.9 (11)	28.1 ± 0.8 (8)	n.d.
D2006A	59.6 ± 7.7 (3)	11.3 ± 1.7 (9)	28.3 ±0.7 (9)	n.d.
D2013A	56.5 ± 9.5 (4)	5.3 ± 0.4 (3)	28.5 ± 1.8 (6)	n.d.
D2014A	54.1 ± 5.6 (6)	4.2 ± 0.6 (13)	27.8 ± 1.5 (4)	n.d.
D2034N	74.8 ± 7.1 (2)	11.0 ± 1.5 (9)	30.3 ± 0.2 (3)	n.d.
E2070A	64.1 ± 2 (6)	11.7 ± 3.7 (3)	25.4 ± 1.1 (4)	n.d.
P2129A	Non-functional			
F2130A	n.d.	4.3 ± 0.7 (4)	n.d.	56.3 ± 1.7 (6)
L2131A	Non-functional			
V2132A	n.d.	9.6 ± 0.6 (3)	n.d.	59.5 ± 1.7 (4)
E2133A	61.1 ± 11.8 (3)	18.2 ± 2.8 (10)	14.4 ± 0.5 (5)	26.8 ± 0.6 (4)
E2133D	85 ± 2.0 (2)	35.8 ± 1.5 (32)	n.d.	77.5 ± 3.0 (5)
E2133Q	74.6 ± 6.2 (5)	29.5 ± 1.8 (26)	n.d.	30.5 ± 1.3 (5)
E2133K	8.1 ± 7.1 (10)	12.2 ± 0.6 (9)	n.d.	20.0 ± 1.3 (4)
L2134A	n.d.	5.3 ± 1.6 (4)	n.d.	59.4 ± 2.1 (5)
R2135A	Non-functional			
D2139A	57.2 ± 6.7 (4)	7.7 ± 0.8 (12)	32.4 ± 1.5 (4)	64.2 ± 1.2 (7)
W2140A	Non-functional			
D2144A	63.0 ± 7.2 (3)	18.3 ± 1.5 (11)	30.6 ± 1.0 (4)	69.6 ± 1.8 (4)
E2156A	57.0 ± 4.3 (4)	6.4 ± 0.4 (14)	26.5 ± 0.9 (5)	n.d.
D2157A	65.0 ± 6.0 (3)	8.8 ± 0.7 (19)	29.7 ± 1.0 (3)	n.d.
E2170A	69.0 ± 1.0 (2)	11.6 ± 1.3 (3)	27.8 ± 0.1 (3)	n.d.
E2172A	75.3 ± 7.0 (3)	8.4 ± 0.9 (3)	24.3 ±0.3 (4)	n.d.
**mPiezo2 mutant**				
E2416K	49.6 ± 4.6 (5)	5.7 ± 0.5 (5)	n.d.	32.6 ± 1.0 (6)

Mean ± s.e.m. (n-number); n.d.: not determined
